# Redefining the battle against colorectal cancer: a comprehensive review of emerging immunotherapies and their clinical efficacy

**DOI:** 10.3389/fimmu.2024.1350208

**Published:** 2024-03-12

**Authors:** Salima Shebbo, Najat Binothman, Manar Darwaish, Hanan A. Niaz, Rwaa H. Abdulal, Jamilah Borjac, Anwar M. Hashem, Ahmad Bakur Mahmoud

**Affiliations:** ^1^ Strategic Research and Innovation Laboratories, Taibah University, Madinah, Saudi Arabia; ^2^ Vaccines and Immunotherapy Unit, King Fahd Medical Research Center, King Abdulaziz University, Jeddah, Saudi Arabia; ^3^ Department of Biological Sciences, Beirut Arab University, Debbieh, Lebanon; ^4^ Department of Chemistry, College of Sciences and Arts, King Abdulaziz University, Rabigh, Saudi Arabia; ^5^ Immunology Research Program, King Abdullah International Medical Research Center, Riyadh, Saudi Arabia; ^6^ Department of Medical Laboratory Technology, Faculty of Applied Medical Sciences, University of Tabuk, Tabuk, Saudi Arabia; ^7^ Department of Clinical Microbiology and Immunology, Faculty of Medicine, King Abdulaziz University, Jeddah, Saudi Arabia; ^8^ College of Applied Medical Sciences, Taibah University, Almadinah Almunawarah, Saudi Arabia

**Keywords:** colorectal cancer, vaccines, immune-checkpoints inhibitors, CAR-T therapy, combinational therapy

## Abstract

Colorectal cancer (CRC) is the third most common cancer globally and presents a significant challenge owing to its high mortality rate and the limitations of traditional treatment options such as surgery, radiotherapy, and chemotherapy. While these treatments are foundational, they are often poorly effective owing to tumor resistance. Immunotherapy is a groundbreaking alternative that has recently emerged and offers new hope for success by exploiting the body’s own immune system. This article aims to provide an extensive review of clinical trials evaluating the efficacy of various immunotherapies, including CRC vaccines, chimeric antigen receptor T-cell therapies, and immune checkpoint inhibitors. We also discuss combining CRC vaccines with monoclonal antibodies, delve into preclinical studies of novel cancer vaccines, and assess the impact of these treatment methods on patient outcomes. This review seeks to provide a deeper understanding of the current state of CRC treatment by evaluating innovative treatments and their potential to redefine the prognosis of patients with CRC.

## 1 Introduction

Colorectal cancer (CRC) is the third most prevalent cancer worldwide. In 2020, CRC was diagnosed in 1.93 million new patients and considered the second leading cause of cancer-related deaths (1). Although CRC mortality rates in different countries have declined over the past few years, survival rates remain substantially low (2). Patients with metastatic CRC (mCRC) have a 5-year survival rate of only 10% (3). Early screenings and improvements in treatment have fortunately contributed to a decrease in the incidence and mortality of CRC. A recent study, however, has found a shift in the incidence of CRC, with more cases diagnosed in younger patients (i.e., under 50 years of age) and those with more advanced stages of the disease (4).

CRC is conventionally treated with laparoscopy, surgery, radiotherapy, and chemotherapy. For decades, neoadjuvant and palliative chemotherapies combined with surgery have been the standard treatments for mCRC (5). However, these interventions exhibit minimal efficacy, and disease relapse due to resistance to chemotherapy is frequent (6). Therefore, alternatives for treating CRC effectively are vital. Interestingly, a paradigm shift occurred in CRC treatment upon the introduction of immunotherapy (7). This modality has gained momentum since immune checkpoint blockade was first approved for treating melanoma (8). Unlike conventional treatments, immunotherapy makes use of patients’ own immune system to fight cancer. It activates innate and adaptive immune responses to combat cancer progression (9). Immunotherapy has shown promising effects on various gastrointestinal cancers, including CRC. Several immunotherapeutic drugs have been approved by the United States Food and Drug Administration (FDA) for treatment (10). These drugs include immune checkpoint inhibitors (ICIs) such as ipilimumab, which targets cytotoxic T-lymphocyte-associated protein 4 (CTLA-4), and pembrolizumab and nivolumab, which target programmed cell death protein 1 (PD-1) (10, 11). These ICIs specifically target T-cell-negative regulatory molecules, thereby alerting the immune system to attack and eradicate the abnormal cells without affecting normal cells (11). The currently approved ICIs, however, have shown to be largely ineffective in the majority of patients with pMMR-MSI-L, which accounts for 95% of all cases of mCRC. Thus, it is imperative that novel treatment strategies be developed for these patients. A number of immunotherapeutic strategies are currently being evaluated, including combinations of ICIs with chemotherapy, VEGF inhibitors, cancer vaccines, adoptive cell transfer and BTC antibodies (12). While some patients do not respond to immunotherapy owing to their condition, others show better prognosis and quality of life (13). Thus, the present review aims to shed light on the potential of different immunotherapeutic approaches for treating CRC, particularly vaccines, ICIs, and chimeric antigen receptor T-cell (CAR-T) therapies. It also discusses the results of clinical trials assessing the efficacy of each therapy in patients with CRC.

## 2 CRC vaccines: potential targets, vaccine types, and combination therapies and promising vaccines in preclinical stage

### 2.1 Exploring potential vaccine targets for CRC: an analytical overview of counteracting mechanisms

Identification of the ideal and correct antigen for a cancer vaccine is a pivotal step in the process of vaccine construction. The antigen must be highly immunogenic, expressed solely on tumor cells or overexpressed in them, and crucial for their survival. The activation of T lymphocytes by tumor antigens upon their binding is imperative in cancer vaccines.

Tumor antigens are proteins classified into tumor-associated antigens (TAAs) and tumor-specific antigens (TSAs). They are commonly identified based on their ability to elicit an anti-cancer immune response, their levels compared to healthy cells, and the type of tissue in which they are found (14). TAAs are proteins released at significantly lower amounts in normal cells compared to cancerous cells (15). However, TSAs are proteins produced solely in tumor cells (15).

Jia et al. reported a number of TAAs and TSAs that have been extensively studied and used in CRC vaccine development due to their potential in escaping immune reactions or promoting cell survival. TAAs in CRC include Carcinoembryonic antigen (CEA) and melanoma-associated antigen (MAGE), mucin 1 (MUC-1), epidermal growth factor receptor (EGFR), vascular endothelial growth factor receptor 1 and 2 (VEGFR1, VEGFR2), transmembrane 4 superfamily member 5 protein (TM4SF5), survivin, mitotic centromere-associated kinesin (MCAK), guanylyl cyclase C (GUCY2C), and 5T4 (16). Additionally, Wagner et al. mentioned some of the TSAs that can be used as vaccine targets in CRC, which are often associated with frameshift mutations in coding microsatellite regions like PTHL3, HT001, AC1, ACVR2, SLC23A1, BAX, TCF-4, and MSH3. Apart from these genes, peptides proposed for studying MSI-H CRC vaccines, produced from frameshift mutated MARCKS-1, MARCKS-2, TGFβRII, TAF1B‐1, PCNXL2-2, TCF7L2-2, Baxα+, CREBBP, AIM2, EP300, and TTK, are determined based on experimentation and bioinformatics data (15). With all this being mentioned, vaccines based on the use of tumor antigens that make these antigens available to APC and activate the immune cycle will induce the infiltration of immune cells to the tumor site and the activation of the immune system against cancer.

### 2.2 Emerging role of therapeutic vaccines in treating and preventing CRC

Over the past decade, cancer vaccines have been extensively studied owing to the availability and cost-effectiveness of sequencing technologies that can identify diverse tumor neoantigens (15). Generally, cancer vaccines include cell-based, virus-based, peptide-based, and nucleic acid-based vaccines (17). Despite the enormous challenges scientists face in designing safe, tolerable, and immunogenic vaccines, many clinical trials have successfully tested vaccines for treating CRC (15, 18). This section describes cancer vaccines that have been used to treat CRC in clinical trials, discussing the approaches and their downsides and possible ways to improve their clinical outcomes.

#### 2.2.1 Cell-based vaccines

In cell-based vaccines, cells are used to stimulate the immune system to attack cancer cells (19). There are two main types of cell-based vaccines: tumor cell-based vaccines and dendritic cell (DC)-based vaccines. **Table 1** summarizes the clinical trials that have been conducted to assess the safety and efficacy of cell-based vaccines in patients with CRC. A phase II study enrolling three patients with CRC and liver metastasis explored the effect of the Vigil™ autologous vaccine, a novel dual-modulatory autologous tumor cell-based vaccine. In this vaccine, cells are transfected with a DNA plasmid encoding a granulocyte–macrophage colony-stimulating factor (*GM-CSF*) transgene and a bifunctional shRNA construct to knock down furin convertase and prevent GM-CSF degradation by Tgfb1 and Tgfb2. In the study, the vaccine was used in combination with folinic acid (leucovorin), fluorouracil (5-FU), and oxaliplatin (FOLFOX-6) chemotherapy (38). Two patients showed a disease-free survival (DFS) of over 8 years after receiving 12 doses of Vigil with FOLFOX-6. This study demonstrated a significant induction of long-lasting systemic adaptive immunity among patients. Vigil, in combination with FOLFOX-6, was found to be safe and exhibited a potential antitumor effect against advanced CRC with resectable liver metastases (38). A clinical trial in patients with advanced cancer, including CRC, also demonstrated the potential of Vigil to induce an immune response that correlates with prolonged survival (20). All of these findings point to Vigil™ as a potential treatment option for people with advanced colorectal cancer that is worth further investigation and development.

**Table 1:** Different vaccines in CRC clinical trials.

Type of Immunotherapy	Status/Country	Route of administration	Clinical Phase	Vaccination Strategy	Combination Therapy	Main Findings	NCT identifier	Ref.
Tumor cell-based vaccines	Terminated (Business Decision to pursue other indications) United States	i.d.	II	Vigil™ autologous vaccine that contains rhGM-CSF transgene and a bifunctional shRNA construct to knockdown furin	FOLFOX-6 (Chemotherapy)	-Patients showed no evidence of disease recurrence for over 8 years. -A systemic immune response to vigil therapy was observed.	NCT01505166	(20)
RNA-pulsed DC vaccine	Completed United State	i.v.	I/II	dendritic cells are taken from patients then pulsed with CEA RNA then reinjected into the patient’s body	NA	- Administering patients with advanced malignancies with mRNA-loaded DC is both feasible and safe	NCT00003433	(21)
Autologous tumor cell vaccine plus BCG vaccine	United State Netherland	i.d.	III	adjuvant active specific immunotherapy (ASI) with an autologous tumour cell-BCG vaccine with surgical resection	ASI BCG vaccine	ASI showed a: -mininal adverse reactions - a significant clinical benefit observed in surgically resected patients with stage II colon cancer	NA	(22)
Autologous tumor lysate with Cytokine-Induced Killer Cells	United state Canada	i.v.	I/II	DC pulsed with autologous tumor lysate combined with CIK	CIK	-Significantly higher levels of IFN-c and IL-12 - Reduced the risk of post-operative disease progression and improved OS	NA	(23)
Autologous tumor lysate pulsed DC and CD40L	United State completed	i.n.	NA	Autologous monocyte stimulated with rhuGM-CSF then cultured with tumor cell lysate and then on day 7 with recombinant human CD40L	CD40L	-Among the responders, 63% exhibited a 5-year RFS rate. -The DC vaccine with CD40L did not result in increased immune responses.	NA	(24)
Therapeutic autologous dendritic cells	United State Completed	s.c. i.d.	II	Patients undergo leukapheresis to obtain autologous DC loaded with: -vaccinia-CEA-MUC-1-TRICOM (PANVAC-V) dendritic cells Then patients receive autologous -fowlpoxCEA-MUC-1-TRICOM (PANVAC-F) vaccine	-Falimarev -Inalimarev -Sargramostim	The Recurrence-free survival (RFS) at 2 years was similar in both arms, namely, (DC/PANVAC and PANVAC/GM-CSF)	NCT00103142	(25)
Therapeutic autologous dendritic cells	United State Completed	-Denileukin diftitox i.v. -Recombinant fowlpox-CEA (6D)-TRICOM vaccine i.d. s.c.	I	Patients receive denileukin diftitox IV over at least 15 minutes -vaccine therapy comprising autologous DC infected with recombinant fowlpox-CEA (6D)-TRICOM	-Denileukin diftitox -Recombinant fowlpox-CEA(6D)/TRICOM vaccine	- Combining Denileukin diftitox with vaccines is safe and effective, with promising results observed in the multiple-dose group, but not in the single-dose group.	NCT00128622	(26)
Mutant ras peptide-based vaccine	United State Completed	s.c.	II	-Patients received 13-mer mutant ras peptide, spanning aa 5– 17 - 250 µg of DETOX - 25 µg of monophosphoryl lipid A (MPL)	-DETOX (cell wall skeleton of Mycobacterium phleia) - MPL from Salmonella Minnesota R 595	-The vaccine is feasible, safe, and has a positive effect on immune response and overall survival.	NA	(27)
Mutated Ras peptide Vaccine	United State Completed	s.c.	II	Arm 1: Patients receive vaccine and Detox pc with IL-2 -Arm 2: patients received Vaccine admixed with DetoxPC sc and GM-SCF -Arm 3: Patients received vaccine admixed with DetoxPC sc with Il-2 and GM-CSF	-IL-2 (aldesleukin) -GM-CSF (sargramostim) -DetoxPC	-Il-2 has a negative effect on the immune response induced by the mutated Ras peptide vaccine. -Highest immune response was seen in Arm 2, where vaccine is combined with GM-CSF.	NCI97C0141	(28)
messenger ribonucleic acid (mRNA)-based vaccine	United State Terminated (slow accrual)	i.m.	I/II	Using tumor-infiltrating lymphocytes (TIL) a specific immunogenic mutations expressed in patients' tumor are identified. -The validated and defined neoantigens, predicted neoepitopes, and mutations of driver genes were concatenated into a single mRNA construct	NA	Safe and induced T cell response against predicted neoantigen.	NCT03480152	(29)
ZYC300	United State Completed	i.m.	I	ZYC300 is a DNA plasmid vaccine administered at least 6 and up to 12 doses in alternating lateral quadriceps at 400 µg DNA/dose once every 2 weeks	NA	-Safe and feasible -Unexpectedly, an association between immunity to CYP1B1 and response to salvage therapy was noticed	NA	(30)
Influenza vaccine	Denmark Completed	i.t.	I/ II	Intratumoral application of an unattenuated influenza vaccine	Curative surgery	-Elevated level of CD8+ T cells infiltration in tumor accompanied by an increase in the transcript expression encoding to cytotoxic activity. - Upregulation of PD-L1 and downregulation of FOXP3.	NCT04591379	(31)
Ad5-hGCC-PADRE vaccine	United State Completed	i.m.	I	-GUCY2C residues 1–429 with a C-terminal PADRE epitope cloned into the E1 region of pAd/CMV/V5 obtaining E1- and E3-deleted human serotype 5 adenovirus	Surgically resected stage I/II	-CD8+T cell and antibody response against self-antigen with no detection of CD4+ T cells.	NCT01972737	(32)
AD5 CEA Vaccine	United State Completed	s.c.	I/II	It is a dose escalating strategy: -Cohort 1: Received 1×109 VP in 0.5 ml subcutaneously (SQ) in the same thigh every 3 weeks for 3 immunizations -Cohort 2: dose of 1×1010 VP in 0.5 ml SQ every 3 weeks for 3 treatments -Cohort 3: dose of 1×1011 in 0.5 ml SQ every 3 weeks for 3 treatments.	NA	-Safe and effective despite the presence of neutralizing antibody against AD-5.	NCT01147965	(33)
VRP-CEA(6D)/AVX701	United State Completed	i.m.	I	4 x 10EE8 IU intramuscularly every 3 weeks for 4 total immunizations	NA	-Safe and effective T cell response was associated with longer survival in stage IV. -The rate of T cell response and antibody response were higher in stage III.	NCT01890213	(34)
Ad-sig-hMUC-1/ecdCD40L vector	Singapore Unknown Recruiting last update in October 2016	s.c. in preclinical studies	I	-Adenovirus vector encodes for a fusion protein in which the hMUC-1 antigen is connected to CD40L (CD40 ligand) -A dose escalating procedure to determine the maximum dose that can be used with no toxicity	NA	-Safe and showed low toxicity. -No dose limiting toxicity and MTD wasn’t reached. -Encouraging anti-tumor activity was noticed.	NCT02140996	(35, 36)
Pexa-Vec	United State	i.v.	I/II	Pexa-Vec is a vaccinia virus with an inactivated thymidine kinase gene that is designed to express hGM-CSF and beta-galactosidase	-Durvalumab -Tremelimumab	-The vaccine is safe and well tolerated -This combination has demonstrated a potential therapeutic efficacy in pMMR mCRC	NCT03206073	(37)

s.c, subcutaneous; i.d, intradermal; i.v, intravenous; i.n, intranodal; i.m, intramuscular.Not Applicable (NA).

Hu et al. reported the outcomes of a clinical trial that enrolled 254 patients with stage II and III CRC to test adjuvant active specific immunotherapy with an autologous tumor cell-bacillus Calmette-Guerin vaccine (OncoVAX^®^). This vaccine comprises irradiated autologous tumor cells with weakened live bacillus Calmette-Guerin as an immune adjuvant to prevent CRC recurrence following surgery (22). This trial was more effective in resectable treated rather than resectable alone. A significantly longer recurrence-free period and a 61% reduction in disease recurrence were observed. Phase III of the clinical trial revealed a notable beneficial effect of OncoVAX on the recurrence-free interval (57.1% relative risk reduction), overall survival (OS; 5 years), and recurrence-free survival (RFS; 5 years) among patients with stage II CRC (39). These results pave the way for new developments and underscore the importance of further research to unravel the potential effects of combining adjuvants with vaccines for enhancing treatment strategies in colorectal cancer.

A phase II clinical trial assessed the effect on disease progression and clinical benefits of autologous tumor lysate-pulsed DC immunotherapy with cytokine-induced killer cells in a small cohort of patients with gastric cancer (GC) and CRC. A total of 46 patients were enrolled in the study, with 14 and 13 patients randomly assigned to the cell-based immunotherapy group and control group, respectively (23). Patients who received cell-based immunotherapy combined with low-dose chemotherapy had higher interferon-gamma (IFN-γ) and interleukin (IL)-12 levels than controls. Additionally, patients who received cell-based immunotherapy had a lower risk of disease progression after surgery (p<0.01) and longer OS (p<0.01). These results suggest that DC/cytokine-induced killer immunotherapy is a promising and effective treatment for GC and CRC. This study emphasizes the value of combining chemotherapy or radiotherapy with DC/cytokine-induced killer immunotherapy, paving the way for further improvements in treatment efficacy (23). Combining immunotherapy with chemotherapy is crucial for treating CRC; however, the dosage plays a pivotal role in determining the outcome of these treatment modalities.

Apart from autologous tumor cell-based vaccines, DC-based vaccines have been extensively tested in preclinical and clinical trials (40). DC-based vaccines are made by taking patients’ DCs and loading them with tumor antigens. Loaded DCs are then injected back into patients to train the immune system to recognize and attack cancer cells (41). At Duke Cancer Institute, Morse et al. evaluated the effectiveness of a carcinoembryonic antigen (CEA) RNA-pulsed DC cancer vaccine and RFS in patients with resected liver metastases from colon cancer (21). The CEA RNA-pulsed DC cancer vaccine used DCs to deliver an RNA encoding the CEA protein. This protein is often found on the surface of cancer cells (42). In this trial, patients underwent leukapheresis, and their cells were then exposed to recombinant human-GM-CSF and recombinant human-IL-4 in a medium to generate DCs. They were loaded with mRNA encoding CEA. This phase I/II clinical trial revealed the safety and possibility of using mRNA-loaded DCs in patients with advanced malignancies (21). Therefore, using the patient’s own dendritic cells loaded with tumor antigen is a safe and practical method that raises the possibility that mRNA-loaded DCs could be used as an effective treatment for advanced cancers. This bolsters the continuous endeavors to utilize the immune system’s potential in combating malignancy.

Another randomized clinical trial in patients with resectable mCRC used autologous tumor lysate-pulsed DCs and CD40L (24). After tumor resection, the tumor was irradiated and lysed in three freeze–thaw cycles in liquid nitrogen. DCs isolated from patients’ own peripheral blood mononuclear cells and transfected with recombinant human CD40L were loaded with tumor lysate to generate autologous tumor lysate-pulsed DCs expressing CD40L. This trial demonstrated increased IFN-γ levels in 15 of 24 patients, indicating T-cell proliferation. The 5-year RFS rate was 63% in responders and 18% in non-responders (p=0.037). This work adds significant knowledge to the expanding corpus of research demonstrating the function of autologous tumor lysate-pulsed DCs in boosting immune responses and maybe benefiting long-term outcomes in patients with resectable metastatic colorectal cancer.

#### 2.2.2 Peptide-based vaccines

Peptide-based cancer vaccines use synthetic peptides to stimulate the body’s immune system to attack cancer cells. They have several advantages over other types. They are relatively easy to produce and can be customized to target specific antigens. However, peptide-based cancer vaccines also have limited effectiveness; thus, they are often combined with adjuvants to improve the overall immune response (19, 43). **Table 1** summarizes several clinical studies assessing the therapeutic efficacy of peptide-based vaccines in treating CRC. A phase II trial demonstrated the safety and feasibility of a 13-mer mutated K-Ras peptide used as an adjuvant vaccine for CRC and pancreatic cancer (44). This peptide is 13 amino acids long, including the most common mutation, G12V (45). The study included five patients with pancreatic cancer, seven patients with CRC, and 12 individuals with no evidence of disease. The 13-mer mutant K-Ras peptide caused an increased IFN-γ mRNA expression in five of 11 patients. The mean DFS was 35.2+ months, and the mean OS was 44.4+ months in patients with pancreatic cancer, whereas the mean DFS was 27.2+ months, and the mean OS was 41.5+ months in patients with CRC (27). Moreover, Rahma et al. combined the mutated K-Ras vaccine with IL-2 and/or GM-CSF to treat solid metastatic tumors, including CRC, to augment the immune response to the vaccine. Their study included 53 patients with colorectal (n=38), pancreatic (n=11), lung (n=3), and common bile duct (n=1) cancers divided into three treatment arms (A: 16, B: 18, and C: 19) (28). The results showed that 92.3% of patients in arm B, 31% in arm A, and 36% in arm C had a positive immune response (p=0.003). Although the vaccine induced an immune response with GM-CSF, it failed to yield a high-rate response when combined with IL-2 regardless of GM-CSF presence. This finding implies that IL-2 has a detrimental effect on the vaccine, and further studies are needed to unravel its unfavorable influence on the immune response rate when combined with the vaccine. Nevertheless, vaccine administration was correlated with an increased DFS and OS. All of these findings add important information to the current discussion on the development of combinatory and targeted immunotherapies for solid metastatic cancers, highlighting the necessity for sophisticated strategies in the search for efficient cancer therapeutics.

In addition to K-Ras, TOMM34 and RNF4 are overexpressed among patients with CRC, making them promising drug targets (15). A phase II clinical study assessed the cytotoxic T lymphocyte (CTL) response to a cocktail of two epitope peptides with uracil–tegafur (UFT/LV) chemotherapy to evaluate its effect on the survival rate as an adjuvant immunotherapy. The study enrolled 44 patients categorized into two groups: 28 into the HLA-A*24:02-matched group and 16 into the unmatched group. In the first group, 14 patients showed a CTL-positive response for RNF43 and/or TOMM34 peptides after two regimen cycles. In the second group, 10 patients showed a similar response. The 3-year RFS rate was significantly higher in the CTL-positive group than in the CTL-negative group (46). Similarly, Hazama et al. tested a cocktail vaccine consisting of five peptides [RNF43-721, TOMM34-299, KOC1(IMP-3)-508, VEGFR1-1084, and VEGFR2-169] in combination with oxaliplatin (FOLFOX, XELOX) in their phase II clinical trial among patients with advanced CRC (47). This study was based on a phase I trial showing that the multiple peptide-based vaccine was safe, with a low risk of systemic adverse reactions (48). The phase II study generated interesting results, including the OS of the HLA-A*24:02-matched group being higher than that of the unmatched group (p=0.032) when patients received the vaccine for more than 1 year. The neutrophil–lymphocyte ratio was also noted as a predictive marker for regimen responsiveness, making it a criterion for choosing eligible patients (47). The vaccine was well tolerated, but the sample size was a limiting factor. Moreover, the fact that immunosuppressive cells such as regulatory T cells (Tregs) enable tumors to escape the immune response suggests that the vaccine must be combined with another drug in future studies to modulate and reduce the immunosuppressive nature of the tumor microenvironment (TME). The phase II results demonstrated the need for a phase III trial for this cocktail vaccine, as it showed effectiveness in a specific patient subset. Further, the neutrophil–lymphocyte ratio and percentage of lymphocytes were confirmed to be predictive biomarkers for treatment responsiveness.

In conclusion, peptide-based vaccines have been shown to be effective against tumor growth and metastasis; however, this effect is robustly observed with cocktail approaches and when peptide-based vaccines are combined with other treatment options.

#### 2.2.3 Nucleic acid-based vaccines

##### 2.2.3.1 mRNA vaccines

mRNA vaccines are formulated *in vitro* to encode and produce tumor antigens that can induce an immune response (49). They induce broad humoral and cellular immune responses and increase the possibility of overcoming resistance to cancer vaccines. **Table 1** shows the results of several clinical studies of nucleic acid-based vaccines provoking an immune response in patients with CRC. An ongoing phase II clinical trial (NCT03948763) is evaluating the safety, tolerability, and optimal dose of an mRNA vaccine (mRNA-5671/V941) that targets four of the most common KRAS mutations (G12D, G12V, G13D, and G12C). Moderna and Merck collaborated to produce the mRNA-5671 vaccine, and an active phase I trial is testing it solely or in combination with pembrolizumab (50). In this trial, mRNA-5671 is delivered intramuscularly within lipid nanoparticles for a total of nine cycles every 3 weeks. As a preliminary outcome, this protocol yielded an antitumor response, and the formulation was well tolerated. When mRNA vaccines are taken up by antigen-presenting cells (APCs), the epitopes of translated peptides are presented by their major histocompatibility complexes (MHCs), leading to the initiation of both CTL- and memory T-cell-dependent immune responses.

Liu et al. reported on various mRNA vaccines that have been tested in phase I or II clinical trials against melanoma and other tumors and have shown promising results, including TriMix, BNT111, mRNA-4157, and BNT122. These vaccines encode immunomodulatory molecules, inflammatory cytokines, and tumor antigens (19). A phase II trial demonstrated a robust CD8^+^ T-cell response using TriMix with a tumor-associated antigen (TAA) mRNA in patients with stage III and IV melanoma. Further, BNT111, a cocktail mRNA vaccine that encodes four TAAs (NY-ESO-1, MAGE-A3, tyrosinase, and TPTE), proved to be a potent immunotherapeutic vaccine for melanoma in combination with a checkpoint inhibitor (51). Thus, mRNA vaccines are emerging as major players in future cancer treatment, opening doors for newer directions in research and clinical applications.

BioNTech and Genentech designed a neoantigen mRNA-based vaccine (RO7198457; NCT03289962) and tested it in phase I clinical trials on various cancer types, including CRC (50), either as a monotherapy or in combination with atezolizumab. The former regimen was well tolerated and induced pro-inflammatory cytokine release and a peripheral T-cell response in most patients. Furthermore, at the time of writing this review, BioNTech is still recruiting patients for a phase II trial to test the effectiveness of RO7198457 in patients with circulating tumor DNA-positive, surgically resected stage II/III rectal cancer or stage II (high-risk)/stage III colon cancer (NCT04486378).

Additional vehicles, such as viruses or cell-based vaccines, can be used to enhance mRNA vaccine delivery. As mentioned previously, a clinical study demonstrated the effectiveness and safety of a CEA RNA-pulsed DC vaccine (21). Regarding the use of a virus as a vehicle, Morse et al. conducted two clinical trials of an mRNA vaccine using a viral vector as a delivery vehicle (AVX701), one on patients with stage III CRC (NCT01890213) and the other on patients with advanced or metastatic CEA-expressing solid tumors (NCT00529984). AVX701 is an alphavirus-based viral replicon particle vaccine expressing a modified version of CEA [CEA(6D)] with possible antineoplastic activity. The vaccine induces CTL immune activity against CEA-expressing tumor cells, where the CEA(6D) mutant (Asn to Asp substitution) causes their enhanced recognition by cognate CD8+ T-cell receptors (TCRs). In the two clinical trials, an alphaviral replicon particle encoding the CEA protein using a self-amplifying mRNA was used. The results of the first trial are still pending and not conclusive, but the second trial showed a 5-year survival of 17% and 75% in patients with stage IV and III cancer, respectively. A CEA-specific humoral response was detected in all patients, and IFN-γ-producing CD8+ granzyme B+ TCM cells surged (50). These findings suggest the tendency of viral replicon particle-CEA toward positive immunomodulation by diminishing Tregs and initiating antigen-specific effector T cells (Teffs).

Finally, mRNA vaccines have been used to treat aggressive, poorly accessible, and metastatic solid tumors, such as CRC and melanoma. These mRNA vaccines are more commonly used in combination with an ICI or a cytokine cocktail to boost their antitumor activity (50).

##### 2.2.3.2 DNA vaccines

DNA vaccines are circular bacterial plasmids that encode tumor antigens to activate tumor-specific immune responses (52). They must be translocated into the nucleus to facilitate the transcription and translation of encoded antigens. After being processed in the cytoplasm, these antigens are presented to CD8+ and CD4+ T cells by MHC I and II to elicit an immune response (52, 53). Generally, antigens encoded by DNA vaccines follow one of three pathways: 1) carriage to cytotoxic CD8+ T cells by MHC I, 2) release by secretory or apoptotic bodies after phagocytosis and processing in APCs and presentation to CD4+ T cells by MHC II, and 3) processing in APCs and presentation to CD8+ and CD4+ T cells by MHC I and MHC II, respectively (52–54). The third pathway occurs when DNA plasmids are directly transfected into APCs. Moreover, DNA cancer vaccines can encode several antigens regardless of their size and have high specificity and safety as well as low production costs. Nevertheless, they have not achieved remarkable therapeutic efficacy in clinical settings because of their limited immunogenicity (55).

Some clinical trials have evaluated the therapeutic effect of certain DNA vaccines on CRC (**Table 1**). Gribben et al. tested the safety and feasibility of ZYC300, a DNA plasmid expressing a biodegradable poly-DL-lactide-coglycolide microparticle encasing inactive carcinogen activator cytochrome P450 1B1 (CYP1B1) that may have an antineoplastic effect on CYP1B1-expressing cells. ZYC300 was studied in a phase I clinical trial including 17 patients with advanced-stage and progressive CRC (30). Five patients received 12 doses, and the rest received only six doses. Three of six patients who developed immunity to CYP1B1 had stable disease. In contrast, 11 patients did not develop immunity; all were unresponsive to salvage therapy, but one experienced disease progression. Conversely, five patients who developed immunity to CYP1B1 were responsive to salvage therapy. These findings suggest a link between the development of immunity to CYP1B1 and responsiveness to salvage therapy, which could be mediated via a priming response to this therapy. Hence, further investigation is needed to unravel this association and determine whether it is immunologically mediated or whether the anti-CYP1B1 response makes the tumor cell or microenvironment susceptible and less resistant.

A phase I clinical trial on different cancer types, including CRC, assessed the safety, feasibility, and tolerability of combining ZYC300 with cyclophosphamide. The study was completed (NCT00381173), but the results were not conclusive. Furthermore, a multicenter, non-randomized, two-arm phase I/II clinical trial evaluated the safety and immunogenicity of a DNA vaccine encoding the *DOM-CAP-1* fusion gene that targets HLA-A*02:01 binding peptide CAP-1 from CEA (CEA605–613) in patients with CEA-expressing CRC (56). Compared with 60% of patients with advanced disease, all patients with measurable disease showed a remarkable immunological response; however, 20% and 58% of them had anti-CAP-1 and CD8+ T cells, respectively. A decrease in CEA production was coupled with improved survival. These findings indicate that DNA vaccination reduces peripheral tolerance in normal and cancerous tissues. Additional large-scale and combination studies are needed, such as those with anti-PD-1 antibodies that are currently underway, to authenticate the results of the reviewed studies and improve vaccine efficacy.

Duperret et al. used a synthetic neoantigen DNA vaccine in a preclinical study and found an antitumor effect against tumor neoantigens (57). The vaccine was designed through the assemblage of multi-epitope strings of neoantigens with MHC I binding in a plasmid. An increase in CD8+ cells or CD8+/CD4+ neoantigen-specific immune responses was detected with cytolytic potential and polyfunctional ability, evident in the expression of the degranulation marker CD107 and the simultaneous release of multiple cytokines [IFN-γ, tumor necrosis factor alpha (TNF-α), and IL-2]. Hence, this engineered DNA vaccine was found to induce a CD8+ T-cell antitumor response in a mouse model that affected tumor survival and progression. Further advancements in this vaccine are warranted.

#### 2.2.4 Virus-based vaccines

Viral vaccines utilize viruses as vectors for treating and preventing tumorigenesis. The immunogenic nature of viruses and the ability to genetically modify them make viruses great vehicles for tumor antigens (58). Recombinant viruses, such as adenovirus, have been used in cancer vaccines and shown to activate innate and adaptive immune responses. They can infect professional APCs, mainly DCs, where they express their transgenes. Subsequently, they induce high-avidity CTLs to target tumor cells (59). Studies have reported a higher immunogenicity of tumor antigens encoded by viral vectors than of antigens administered with adjuvants (60, 61). This may be caused by a virus-induced pro-inflammatory response. While the production of recombinant viruses is easy compared with other cancer vaccine strategies, some vectors exhibit disadvantages in terms of triggering the release of vector-specific neutralizing antibodies (NAbs) by the host (62). Viruses are used as vectors/vehicles to deliver TAAs into cells or selectively kill tumor cells, as in oncolytic viruses, to strengthen the immune system and produce a robust immune reaction against tumor cells. One of the most commonly used oncolytic viruses in cancer vaccines is adenovirus owing to its ability to selectively kill tumor cells and induce immunostimulation that overcomes the immunosuppressive nature of the TME (63).

Clinical trials on virus-based vaccines for CRC are summarized in **Table 1**. An exploratory phase II clinical trial evaluated the safety and efficacy of an intratumoral influenza vaccine as an additive treatment in patients with early-stage CRC before curative surgery. The results showed that after immunization, CD8+ T-cell infiltration into tumor locations increased. Additionally, the expression of genes linked to neutrophils was markedly reduced, while transcripts linked to cell-killing activities increased. Spatial protein analysis revealed a significant drop in FOXP3 and a considerable increase in programmed death ligand 1 in specific regions (NCT04591379) (31). The clinical trial was conducted based on the results of a prospective study involving 5146 patients who received the influenza vaccine 1 year before and 6 months after curative surgery. In another study, Gögenur et al. noticed a decreased risk of recurrence in patients who received the influenza vaccine 6–12 months before the intended surgery (64). In contrast, no link between the vaccine and overall mortality or DFS was noted. Additional clinical studies are needed to unveil the reasons behind the oncological outcome of the influenza vaccine opening the door to wise clinical decisions and possible improvements in the treatment of CRC.

Further supporting the abovementioned results is the preclinical study by Newman et al. demonstrating the antitumor efficacy of the vaccine in lung tumors. The results showed that intratumoral injection of unadjuvanted influenza decreased tumor growth (65). This outcome was achieved by converting the cold TME to a hot, immune-infiltrated TME by boosting DCs and CD8+ T cells, specifically by targeting tumor antigens. Furthermore, the vaccine enhanced the efficacy of ICIs by priming patients to respond to them. This study also found that intratumoral injection of the influenza vaccine could provide protection from subsequent active lung infections.

**Figure 1:**
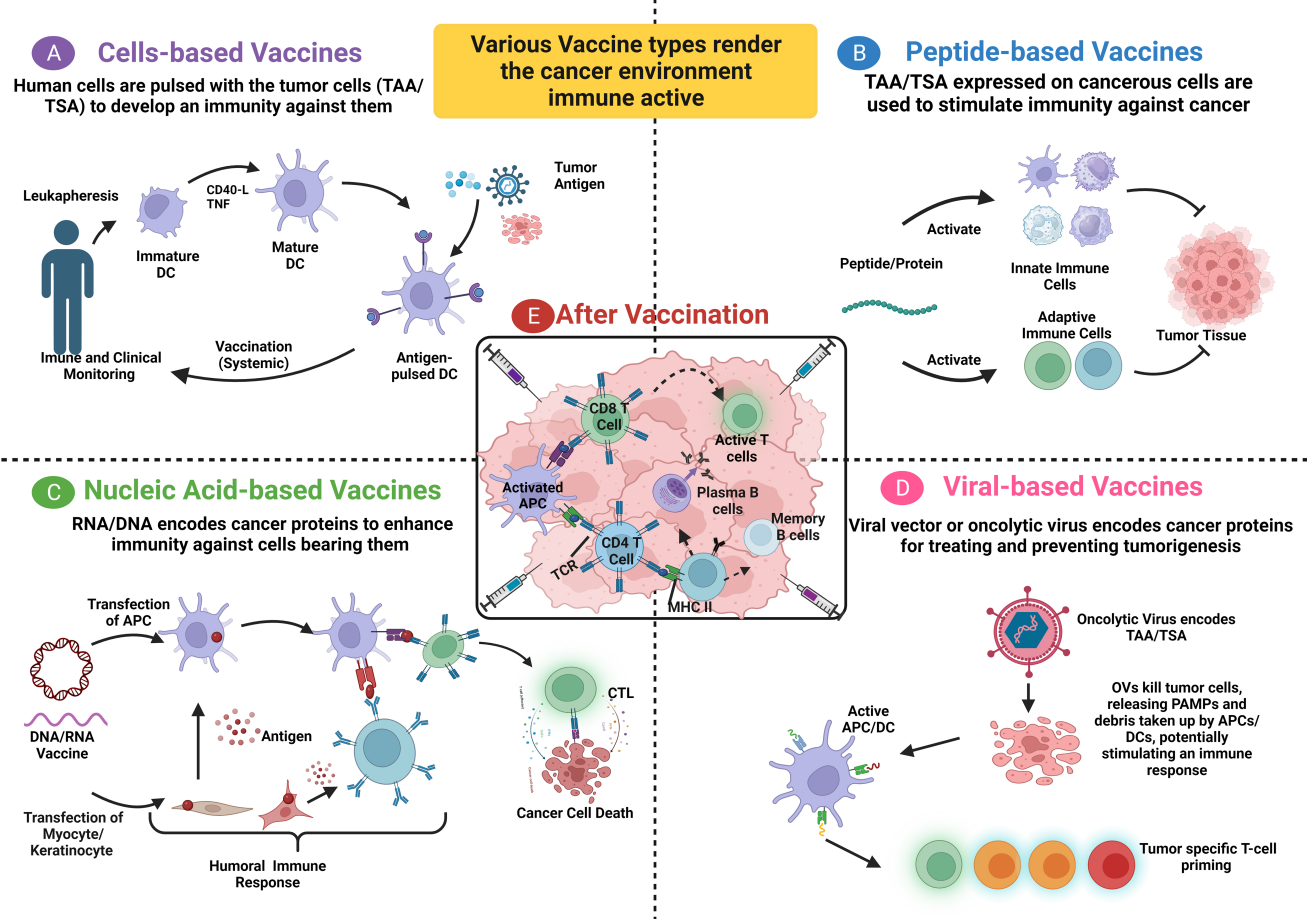
Summary of the four types of vaccines with their mode of action. **(A)** Patients’ blood cells are removed through leukapheresis, and dendritic cells are isolated and expanded from their precursors. These cells are treated with autologous tumor lysate, pulsed with tumor antigens, or engineered with a viral vector, leading to the expression of tumor-associated antigens (TAA) on MHC molecules. Afterward, the antigen-loaded DCs undergo quality check and expansion before being re-infused into the patient’s body. **(B)** A peptide/full-length protein vaccine relies on utilizing TA or a group of such tumor antigens (Cocktail) to activate both innate and adaptive immune cells against tumor cells expressing these antigens. **(C)** DNA/RNA encoded antigen can be transfected into keratinocytes or myocytes using exosomes or apoptotic bodies. Subsequently, the derived peptides and proteins are released and taken up by DC. In parallel, DC can be directly transfected to endogenously express the TA on both MHC I and MHC II, activating T cells to CTL and eliminating tumor cells. A humoral immune response is induced when B cell receptor recognizes protein antigens from somatic cells. **(D)** PAMPs and DAMPs accumulate following the induction of immunogenic cell death in tumor cells through viral oncolysis. Consequently, these PAMPs and DAMPs activate DC, which in turn, activate CTL causing a T cell cytotoxic activity against TAA/TSA, respectively. **(E)** After vaccination, APC cells present tumor antigens to both B cells and T cells, initiating their activation. Activated T cells transform into cytotoxic CTL to target and eliminate tumor cells, while activated B cells differentiate into plasma cells and memory cells. DC, Dendritic cell; TA, Tumor antigen; CTL, Cytotoxic T Lymphocyte; PAMPs, Pathogen-associated molecular patterns; DAMPs, Danger-associated molecular patterns. This figure is created with BioRender.com.

In patients with stage I/II colon cancer, Snook et al. assessed the safety and tolerability of the Ad5-GUCY2C-PADRE vaccine and its ability to induce humoral and cytotoxic immune responses (32). Ad5-GUCY2C-PADRE is a replication-deficient human type 5 recombinant adenovirus (Ad5) vaccine that encodes guanylyl cyclase C (*GUCY2C*) fused to the pan DR epitope (PADRE). Under normal conditions, only intestinal epithelial cells and a subset of hypothalamic neurons have the paracrine hormone receptor GUCY2C, which generates the second messenger cyclic GMP; however, all primary and metastatic human CRCs overexpress GUCY2C. In the study, immunization with GUCY2C-based vaccines generated memory CD8^+^ T-cell responses that provided durable protection against metastases (32). No adverse events higher than grade 1 were noted, and the vaccine induced an immune response skewed to CD8+ T cytolytic cells and antibodies against the GUCY2C antigen. However, no CD4+ T-cell helper response was detected. The split tolerance seen upon Ad5-GUCY2C-PADRE vaccination implies the vaccine’s safety and the importance of this course in molding the body’s immune response against self-antigens. The results emphasize the outcome of a preclinical study showing a split tolerance and significant induction of B-cell and CD8+ T-cell responses (66). Moreover, pre-existing NAbs to the Ad5 vector were noted to negatively influence patients’ immune response to the vaccine, indicating a negative correlation between NAbs and the anti-GUCY2C immune response (32). Additionally, the vaccine is suggested to have an antitumor effect in patients with colorectal, gastric, esophageal, and pancreatic cancers, wherein GUCY2C is overexpressed. The production of self-antigen-independent T cells is pivotal for immunotherapies and needs further investigation to enhance vaccine efficacy and evade helper CD4+ T-cell tolerance. Furthermore, Morse and colleagues evaluated the safety and immunogenicity of the oncolytic adenovirus Ad5 [E1-, E2b-]-CEA(6D) or ETBX-011, manufactured by Etubics Corporation, in patients with CRC (33). ETBX-011 is an adenoviral cancer vaccine formed by manipulating the epitope of human CEA genes inside a replication-defective and E1- and E2b-deleted oncolytic Ad5 virus. Cell-mediated immunity best describes its action, wherein immune cells recognize CEA-expressing cells, empowering T cells to strike against them. This vaccine could also induce an immune response despite the pre-existence of NAbs against adenovirus. Morse and colleagues hypothesized that if the vaccine is effective, the body will develop a robust immune response against tumor cells overexpressing CEA after exposure to the mutated CEA encoded by the virus. Their results showed that the vaccine was safe, induced CEA-specific cell-mediated immunity in most patients despite the pre-existing Ad5 immunity seen in 63% of patients, and increased the OS for 12 months in 48% of patients. This study proved that Ad5 [E1-, E2b-]-CEA(6D) was effective and safe; however, it was performed in a small cohort. Thus, an extended evaluation phase I/II clinical trial was conducted to assess the long-term OS and immune response based on the number of cytolytic T cells and Treg–Teff cell ratio (67). It was deduced that additional booster immunizations are needed to maintain a high level of CEA-directed cell-mediated immunity, as a decreased peak value was noted in five patients. In patients showing strong cell-mediated immunity, high CD4+ and CD8+ T-cell levels were detected. A decreased Treg–Teff cell ratio was also noted in three of five patients. A randomized, controlled phase IIb study will be performed to evaluate the Ad5 [E1-, E2b-]-CEA(6D) vaccine as a booster relative to OS and immunogenicity. Future studies among patients with newly resected early-stage CRC may assist in determining the clinical advantages of this vaccine as an adjuvant. Multitargeted recombinant Ad5 vaccines have also been studied recently and may offer promising outcomes, such as those observed by Bilusic et al. (68). Their phase I clinical trial indicated the safety of a multitargeted recombinant Ad5 PSA/mucin-1 (MUC-1)/brachyury-based vaccine for metastatic castration-resistant prostate cancer. A dose of 5×10^11^ VP was determined for use in a phase II clinical trial, and its use in combination with other immunotherapeutic agents or conventional therapies was suggested. In an extended follow-up phase I/II study in patients with mCRC who received the previously described AVX701 vaccine, Morse et al. evaluated the long-term survival and T-cell and antibody responses in a newly immunized cohort with stage II CRC (34). The results indicated a positive correlation between T-cell responses and prolonged survival in patients with stage IV CRC. The antibody and T-cell response rates were higher among patients with stage III CRC, reflecting a low immunosuppressive environment. Further studies combining AVX701 with ICIs could improve the therapeutic efficiency in a highly immunosuppressive milieu. **Figure 1** summarizes all the vaccine types with their modes of action.

To date, there is no FDA-approved oncolytic virus-based vaccine against CRC despite all the in-depth research in this domain. This is due to many obstacles that need to be overcome to ensure a desired result. These hurdles include the body’s immunity against the virus used and the accurate conveyance of the virus to the target location. On the other hand, the anti-tumor effect of this modality is anticipated to be enhanced through the use of stem cells and immune cells as a delivery platform is underway.

Vaccines encoding tumor antigens have truly revolutionized immunotherapy for treating advanced metastatic CRC. By delivering the tumor antigen, exposing it, and making it accessible to T-cells, a cascade of events occurs: T-cells are primed and activated against the tumor, disrupting the immunosuppressive nature of CRC and rendering the tumor microenvironment the immunologically active. It is paramount to underscore the ongoing significance of delving into additional tumor antigens, particularly tumor-specific ones. This exploration not only holds the potential to enhance personalized cancer immunotherapeutic modalities but also serves as a predictive marker for tumor survival prognosis. Moreover, unraveling the intricate anti-tumor effects of these antigens at the molecular level and understanding the underlying mechanisms will undoubtedly propel the development of cancer therapies, ensuring their utmost effectiveness.

CRC vaccine modalities have exhibited promising results, becoming a pivotal aspect in the search for potent therapy. However, due to the immunosuppressive nature and evasion mechanisms inherent in CRC tumors, treating this type of cancer with monotherapeutic strategies may not yield the desired outcome. Consequently, recent advancements have shifted focus towards combining anticancer vaccines with monoclonal antibodies (mAbs), opening new era of combinatorial curative strategies.

### 2.3 Advancing therapeutic horizons in CRC: efficacy of combinatorial approaches and promising preclinical studies

#### 2.3.1 Therapeutic efficacy of CRC vaccines combined with mAbs

In the last 10 years, mAb therapy has been considered one of the most promising therapeutic approaches for CRC owing to its target specificity. This therapy, alone or in combination with other therapeutic modalities such as radiotherapy or chemotherapy, increases therapeutic potency and reduces toxicity (69, 70). Given that the number of CRC cases is growing, new research frameworks are focusing on creating enhanced mAb and cancer vaccine combinations (71).

Recently, combination therapy utilizing anticancer vaccines and mAbs has emerged as a promising therapeutic approach for CRC. Nucleic acid-based vaccines paired with antibodies are presented as a contemporary strategy among many promising combinatorial curative methods for CRC. For instance, a recent immunotherapeutic approach for CRC used DC mRNA vaccines and bispecific antibodies (70). Another study demonstrated that combining humanized anti-TM4SF5 mAb and TM4SF5-specific peptide-based vaccine can strengthen their anticancer impact and reduce the metastatic potential of colon cancer *in vivo* (72). Hoffmann and colleagues evaluated the use of cetuximab alone or in conjunction with measles virus fusogenic membrane glycoproteins H and F expressed by the HSV-1 vector (70). The authors concluded that the expression of measles virus fusogenic membrane glycoproteins H and F improved cetuximab cytotoxicity and effectiveness by inducing cell–cell fusion. Additionally, mAbs can be paired with CAR-T cells to treat cancer in a novel manner with minimal toxicity and side effects (73).

#### 2.3.2 Promising preclinical studies of novel vaccines for CRC

The aforementioned clinical trials of CRC vaccines highlight the importance of considering the engineering of APCs, potential toxicity of TAAs, pharmacodynamics and pharmacokinetics of designed vaccines, and patients’ immune responses (19). Thus, novel strategies to overcome immunosuppression and immune tolerance and successfully introduce cancer vaccines into the wide array of market drugs are warranted (60). This requires a better understanding of TAAs, the TME, tumor escape processes, and host–tumor interactions to increase the effectiveness and safety of cancer vaccines (15, 18, 19, 74–77).

This section presents some novel cancer vaccines that have shown promising preclinical outcomes in CRC treatment. These vaccines include protein-based vaccines against self-antigens, multi-epitope-based vaccines, immune subtype (IS)-based mRNA vaccines, multitarget chimeric virus-like particles (VLPs), self-adjuvanting and oncolytic vaccines, exosomes, and immunopeptidomes.

Belnoue et al. reported the efficacy of a novel protein-based vaccine (KISIMA) that targets the achaete-scute family bHLH transcription factor 2 (78). This TAA was identified as a promising target for immunotherapy because it is minimally expressed in normal cells. In the preclinical study involving mice with sporadic CRC, the combination of the protein-based vaccine with anti-PD-1 treatment resulted in remarkable tumor-specific immunity and prevented the formation of adenomas. These findings suggest that achaete-scute family bHLH transcription factor 2 is a potential target for immunotherapy in individuals at a high risk of developing CRC.

Corulli et al. designed multi-epitope-based vaccines to prevent and treat CRC by targeting TAAs (CDC25B, COX2, RCAS1, and FASCIN1) associated with a poor disease prognosis (79). In an azoxymethane-induced CRC model and adenomatous polyposis coli mice, immunization with CDC25B- and COX2-based vaccines, but not with RCAS1- and FASCIN1-based vaccines, significantly suppressed colorectal tumors compared with controls, whereby treated mice developed a significantly lower number of tumors in both CRC models. These results indicate the potential of multi-antigen vaccines as a treatment option for CRC.

Liu et al. identified six promising tumor antigens for designing efficacious mRNA vaccines to treat CRC based on the IS (80). These antigens included thrombospondin 2, follistatin-like 3, troponin T1, biglycan, collagen triple helix repeat-containing 1, and NADPH oxidase 4 owing to their association with a poor prognosis and APC infiltration in CRC. Classifying patients according to four ISs characterized by distinctive TMEs showed that IS2 and IS4 yielded significantly enhanced OS and greater immune cell infiltration than did IS1 and IS3. These findings indicate a complex immune landscape that may guide the design of novel mRNA vaccines to treat CRC based on defined ISs.

VLPs have been reported as a platform for cancer vaccines because they display various epitopes and trigger an immune response against tumor cells (81). In their study, Donaldson et al. designed chimeric VLPs as non-infectious, non-replicative subunit vaccines against CRC (82). The recombinant VLPs were made up of rabbit hemorrhagic disease virus VP60 capsid proteins and epitopes from murine survivin and topoisomerase IIα. With a murine model of subcutaneously injected colorectal tumors, the chimeric rabbit hemorrhagic disease virus VLP was found to significantly enhance OS in mice with CRC. The VLPs expressing both survivin and topoisomerase IIα induced a more prolonged remission than did individual monotherapies. Thus, multiple epitopes may enhance therapeutic vaccination in patients with CRC.

Given the role of oncolytic virus-based vaccines in overcoming resistance to ICIs, Das et al. assessed the therapeutic potential of the combination of the self-adjuvanting protein vaccine KISIMA and recombinant oncolytic vesicular stomatitis virus pseudotyped with LCMV-GP expressing TAAs (83). The administration of the combination therapy in a heterologous prime-boost regimen with a well-defined schedule and route of administration in different mouse models of CRC enhanced cancer immunity compared with the components’ individual effects. The combination therapy also significantly altered the TME and elicited an immune response evidenced by the recruitment of persistent antigen-specific cytotoxic T cells. Moreover, the use of heterologous vaccination and ICIs further enhanced the therapeutic outcome regarding long-term survival, suggesting the ability to sensitize non-inflamed tumors to ICIs.

Cell-free vaccines using exosomes have shown promising preclinical results against CRC. They are nano-vehicles released from diverse cells and are essential for cancer initiation and progression. Interestingly, exosomes can alter the behavior of recipient cells based on their cargo. Thus, several studies have attempted to load exosomes with various cargos, including DNA, mRNA, miRNA, and proteins, subsequently eliciting different signaling pathways (84–86). In CRC, Lugini et al. showed that exosomes released in colorectal mesenchymal stromal cells were implicated in CRC progression, angiogenesis, and metastasis (87). These exosomes were shown to overexpress CEA, induce umbilicated spheroids, and release the angiogenic factor miR-210. Consequently, exosomes were suggested as therapeutic tools for treating CRC. For example, Cho et al. studied the therapeutic potential of Hsp70-enriched exosomes in murine models of CRC (88) and found that these exosomes increased MHC II expression and Th1-mediated immune response in tumor cells, indicating a high therapeutic capacity of exosomes in generating tumor regression *in vivo*.

In a similar context, the phase I clinical trial by Dai et al. evaluated the therapeutic efficacy of CEA-containing ascites-derived exosomes combined with GM-CSF in 40 patients with CRC (89). After receiving four weekly subcutaneous immunizations, patients did not develop adverse events and showed a strong tumor-specific CTL response, suggesting the effectiveness of the vaccine in treating mCRC. Thus, exosomes appear to hold promise as cancer vaccines for the treatment of CRC, but further studies are needed to confirm their efficacy and consequently introduce them to clinical settings.

Recently, Jaeger et al. highlighted the importance of profiling MHC I-associated peptides, known as immunopeptidomes, to better understand cancer-related patterns of antigen presentation (90). Upon engineering an affinity tag into an MHC I gene (*H2-K1*) and targeting it to a mouse model of lung adenocarcinoma, the authors could isolate MHC I peptides and profile the immunopeptidome in the disease. The observed differential presentation of peptides in lung adenocarcinoma was not previously reported via mRNA expression or translation efficiency, possibly owing to post-translational processes. The authors further used these peptides as cancer vaccines *in vivo* and observed a significant CD8+ T-cell response in tumor-bearing mice. Their findings suggest reconsidering antigen prediction strategies based on the immunopeptidome, as several cancer-specific peptides minimally express the cognate mRNA. Thus, it may be used in other cancer types and aid in improving the development of peptide-based cancer vaccines.

## 3 Gene-modified T cell therapy

### 3.1 CAR-T therapy: biological aspects and clinical trials

**Figure 2:**
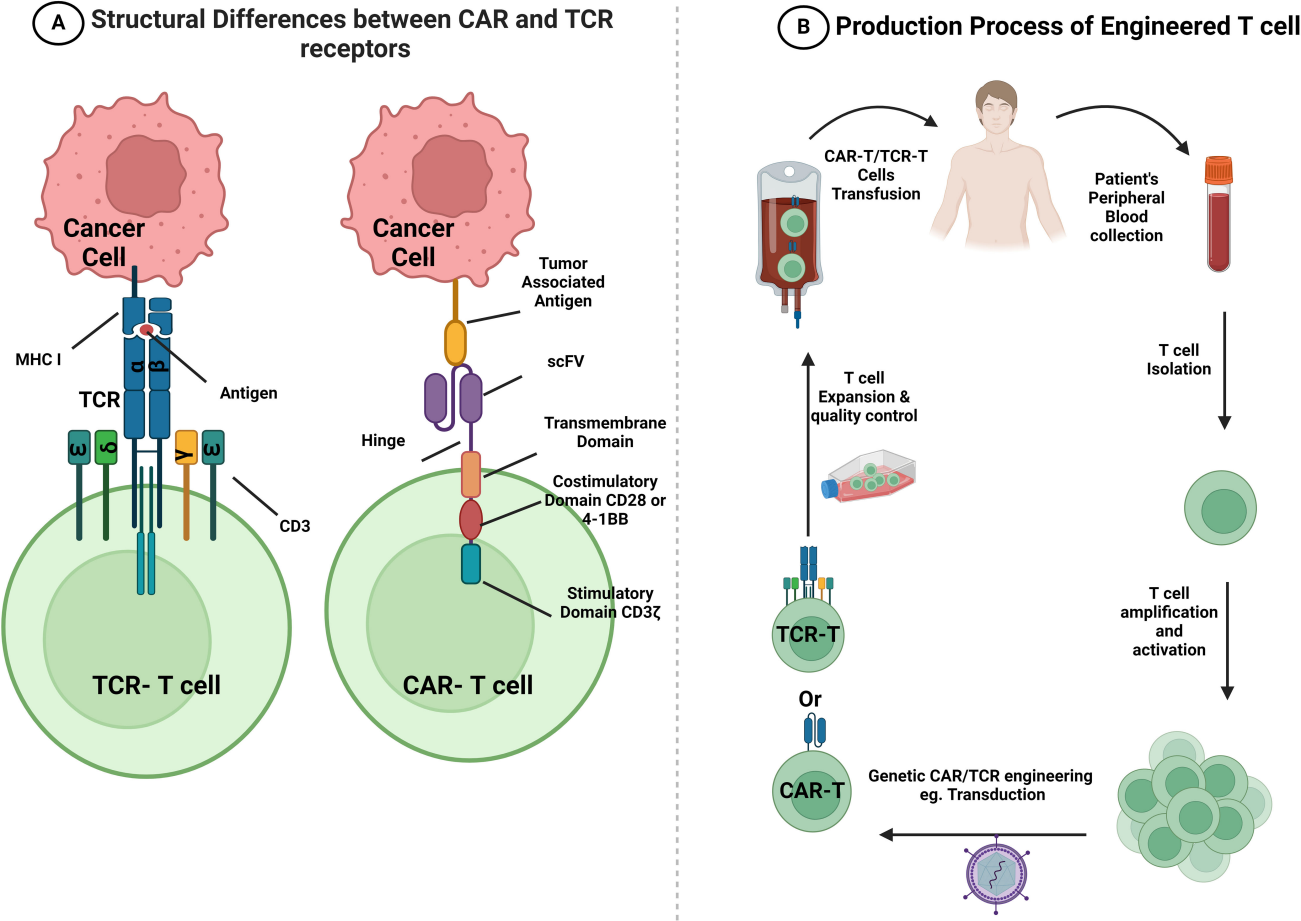
Structural differences between CAR and TCR-T receptors and a summary of CAR-T and TCR-T cell production. **(A)** The CAR receptor encompasses an extracellular antigen-binding domain (scFv domain) that is responsible for binding to TAA on the surface of tumor cells. It is followed by a hinge region, a transmembrane domain, and an intracellular signaling domain. The intracellular signaling domain includes CD3ζ and different co-stimulatory molecules that start an antigen-specific response. However, TCR is a heterodimer which comprises an α and β subunits. These subunits recognize and bind to antigens represented on MHC class I molecules, and they activate T cells through the complex that they form with various CD3 signaling subunits (CD3ϵγ, CD3ϵδ, and CD3ζζ). **(B)** A brief flow chart that visually depicts the sequential steps involved in the process of T cell engineering. Patient’s PBMCs are collected, and T cells are purified from them. Subsequently, T cells are activated and transduced or transfected using a viral vector, such as lentivirus transfection or retrovirus, to display specific CARs or TCRs on the cell surface. After amplification and quality control, CAR-T or TCR-T cells are infused into the patient’s body with the goal of enhancing their anti-tumor ability. CAR, chimeric antigen receptor; TCR, T cell receptor; scFv, single-chain variable fragment; MHC, major histocompatibility complex. This figure is created with BioRender.com.

The advancement of basic research on CAR-T immunotherapy is driven by ongoing work. Numerous prospective CAR-T therapeutic approaches have demonstrated efficacy in preclinical models and early-phase clinical studies for CRC treatment. The main aim of CAR-T therapy is to identify the ideal target or ideal combination of novel checkpoint inhibitors or monoclonal antibodies (mAbs). This approach was designed to widen the spectrum of possible treatments for patients with CRC that could deliver sustainable clinical benefits. CAR-T therapy represents a new era in cancer immunotherapy. In this approach, T cells are extracted from the blood of patients and genetically modified to express a particular chimeric receptor before being reinfused, providing patients with meticulous, exclusive, and individualized therapy. The approach was first developed in 1989 and is considered revolutionary, as it has set up significant safe effects and durable clinical feedback (91); however, it has considerable side effects, including the cytokine release syndrome (92). CAR-T therapy aims to generate functional chimeric receptors that can recognize tumor antigens but not normal antigens in a non-MHC-restricted manner, hinting at the prospect of creating TCRs with any required specificity (93). CAR-T immunotherapy exhibits better selectivity and cytotoxicity via major MHC molecules through the addition of a single-chain variable fragment (scFv) to the TCR than does conventional cell-mediated treatments (94, 95). The CAR construct consists of three domains: 1) a tumor-targeting domain on the scFv that supports T cells in binding to the target antigen on the cell surface (96); 2) a hinge or spacer domain linking the scFv to the transmembrane domain, whose primary function is to increase the flexibility of the scFv and facilitate easy attachment to the target (97, 98); and 3) a transmembrane domain that unites the extracellular and intracellular components, conferring effectiveness and constancy to CAR-T. CD3ζ, CD28, and CD8α are the membranous domains (99) (**Figure 2A**). Apart from the three domains, each CAR contains signaling and costimulatory domains. These costimulatory molecules enhance CAR-T-cell proliferation and persistence, and CD3ζ acts as a T-cell-activating intracellular signaling molecule. The use of CAR-T therapies is increasing along with the number of clinical trials on the subject. As of April 2023, the FDA had endorsed six CAR-T therapies, all authorized to treat blood malignancies but not CRC (100). Although CAR-T therapy is one of the most promising approaches to the adoptive cell treatment of CRC, clinical investigations are still in the early stages. In this section, we describe the possible targets of CAR-T therapy for CRC, with their corresponding expression profiles and clinical studies. Notably, all data regarding clinical trials of CAR-T therapy for CRC were collected from ClinicalTrials.gov; thus, only ClinicalTrials.gov-registered trials were included. Human epidermal growth factor receptor 2 (HER2), epithelial cell adhesion molecule (EpCAM), and mesothelin (MSLN) antigens along with NK group 2 member D ligand (NKG2DL), MUC-1, and CD133 have been licensed for use in clinical trials (**Table 2**) because they are among the most overexpressed antigens in patients with CRC (107). **Figure 2B** provides a concise depiction of the T cell engineering process, illustrating the formation of CAR and TCR-T cells.

**Table 2:** CAR-cells in clinical trials in CRC.

Type of immunotherapy	Status/Country	Clinical phase	Vaccination strategy	Combination therapy	Main Findings	NCT identifier	Ref
HITM-SURE Anti-CEA CAR-T	Complete/ United States	I	-Autologous PBMCs isolated by leukapheresis, then activated with anti-CD3 antibody for 48 hours. -Post-activation, cells were retrovirally transduced with a construct encoding an anti-CEA scfv-CD28/CD3ζ CAR. -Intravenous infusion (IL-2) (50,000 IU/kg/day) for 4 weeks during the CAR-T infusion period.	Interleukin-2 (IL-2)	-Safe and effective. -23.2 months survival time -Marked fibrosis in the liver tumor specimen while the integrity of the normal liver tissue was preserved. -The tumor microenvironment shifted towards a less immunosuppressive milieu, -The recurrent disease emerged in the liver.	NCT02850536	(101)
HITM-SIR Anti-CEA CAR-T	Complete/ United States	I	-Autologous PBMCs isolated by leukapheresis, then activated with anti-CD3 antibody for 48 hours. -Post-activation, cells were retrovirally transduced with a construct encoding an anti-CEA scfv-CD28/CD3ζ CAR.	-Selective Internal Radiation Therapy (SIRT) -Interleukin-2	-Well tolerated -No grade (G) 4/5 events - no instances of severe cytokine-release syndrome (CRS) or neurotoxicity - Reduced levels of GM-CSF-R, IDO, and PD-L1 were detected -The median overall survival time is 8 months	NCT02416466	(102)
Anti-CD133-CAR	Complete/ China	I & II	-CAR T cells produced by directly adding anti-CD3 monoclonal antibody OKT3 to whole PBMCs suspended in culture medium containing interleukin (IL)-2 -Lentivirus-mediated CAR transduction was done on day 3 of cell culture. -After transduction, cells were expanded ex vivo in the presence of IL-2 added three times weekly until the specified cell dose achieved.	CART-EGFR therapy and *anti*-PD-1 antibody	-Safe and effective -The 3-month disease control rate was 65.2% -Median progression-free survival was 5 months. -Repeated cell infusions provide a longer period of disease stability. -NO detectable *de novo* lesions -Feasibility with controllable toxicities, and effective activity	NCT02541370	(103, 104)
Anti-CEA CAR-T Cells	Unknown/ China	I	-Peripheral blood was collected from patients, and PBMCs were isolated. -PBMCs were activated by immobilized CD3 and CD28 antibodies. -Then T cells were infected with lentiviral vector in plates with polybrene. -After viral transduction, T cells were expanded by IL-2 for approximately 12–14 days to		-Promising efficacy -Stable disease after treatment with CEA CAR-T cells -No CAR-related toxicity.	NCT02349724	(105)
EPCAM CAR-T	Recruiting/ China	I	-PBMCs cultured and activated with CD3 antibodies and interleukin (IL)-2 for 24 h. -T cells were transduced with the concentrated lentiviral. -Transduced cells were cultured with IL-2 for 14 days	Radiofrequency/microwave ablation	-Significant increases in cytokine levels while circulating–tumor cells (CTC) in the blood decreased to 0 between 7 days and 4 weeks post-infusion. -No grade 3 or greater hematologic toxicity. -No dose-limiting toxicities (DLT) were reported. -No cases of immune effector cell-associated neurotoxicity syndrome (ICANS) were reported.	NCT05028933	(106)
CEA CAR-T cells	Recruiting/ China	I	NA	Pretreatment with Fludarabine and Cyclophosphamide	Final data collection for the primary outcome measure is due on May 15, 2024	NCT05396300	NA
Autologous CAR-T/TCR-T Cell	Recruiting/ United states	I & II	NA	Pretreatment with Fludarabine and Cyclophosphamide	Final data collection for the primary outcome measure is one to March 1, 2023	NCT03638206	NA

Not Applicable (NA).

#### 3.1.1 HER2, EpCAM, and MSLN

*HER2* is an oncogene that encodes transmembrane glycoprotein receptors. Under normal conditions, HER2 ruffles to the cytosol, where it acts as an intracellular tyrosine kinase (108). Recent studies have demonstrated the effectiveness of HER2-targeted CAR-T therapy against HER2^+^ tumor cells, leading to shrinkage of tumors, elimination of CRC xenografts, protection against recurrence, and increased survival benefit in comparison with control (109). Although HER2 is a promising target for treating malignancies, further assessment of its role in CRC is required. For instance, a phase I/II clinical study (NCT02713984) was withdrawn owing to safety considerations and the reforming of the CAR structure. Another phase I clinical study (NCT03740256) is ongoing. In this clinical trial of solid tumors such as CRC, two combinations of CAdVEC (oncolytic virus) and HER2-specific autologous CAR-T are being assessed for their survival in patient blood and effect on tumor cells. Primary outcomes are expected in December 2024. Notably, these therapies are not yet approved by the FDA. EpCAM, another antigen that has been tested, is regarded as a novel target for adoptive T-cell treatment and a possible emerging biomarker for circulating tumor cells (110, 111). EpCAM CAR-T cells show lytic cytotoxicity against target cells and secrete cytotoxic cytokines such as TNF-α and IFN-γ in an EpCAM-dependent manner. These engineered CAR-T cells greatly reduce the genesis and progression of tumors in xenograft mouse models (112). In addition to HER2 and EpCAM, MSLN is another candidate target. MSLN is a 40-kDa GPI-anchored protein expressed in solid tumors (113). A recent study used MSLN to target various solid cancers, including CRC, and found that MSLN CAR-T cells killed not only MSLN-positive cancer cells *in vitro* but also MSLN-positive CDX and PDX solid tumors *in vivo* (114). MSLN CAR-T cells represent a potential breakthrough in the treatment of solid tumors, but more clinical trials are needed to evaluate their efficacy in treating MSLN-positive CRC tumors.

#### 3.1.2 NKG2D

Clinical trials of NKG2D CAR-T cells that target NKG2DL have been conducted. NKG2D-based CAR-T therapy has demonstrated dose-dependent cytotoxicity against CRC cells, strongly inhibited tumor growth, and increased overall mouse survival (115). In a phase I study (NCT03310008), SHRINK, an NKG2D CAR-T cell construct comprising the CD8α signal sequence with an external portion of the human NKG2D receptor (amino acids 82–216), spacer region of CD8α, transmembrane and intracellular domains of CD28, and internal signaling component of human 4-1BB and CD3ζ, was constructed with two restriction sites for EcoRI and BamHI at both ends. The parental minicircle plasmid, pPMCCMV-MCS-EF1-GFP-SV40polyA, was used to clone the entire gene sequence. This plasmid was then transformed into *Escherichia coli* ZYCY10P3S2T minicircle-producing strain to create a minicircle vector. The novel minicircle DNA vector was designated the KG2D CAR minicircle DNA vector. Maher and Davies conducted a phase I dose-escalation clinical trial, in which this construct was administered concurrently with FOLFOX chemotherapy in patients with CRC and liver metastasis (NCT03018405) (116). The primary results revealed its safety, with no dose toxicity limitations. Furthermore, Deng et al. demonstrated that NKG2D CAR-T cells exhibited specific cytotoxicity in human CRC cell lines, with promising immunotherapeutic activity (115).

#### 3.1.3 CEA

*CEA*, a common tumor marker in CRC, has also been used in CAR-T therapy. Preliminary research on CAR-T therapy targeting CRCs with liver metastases expressing CEA revealed its potential in preventing immunosuppression (117). The NCT02349724 phase I trial tested CEA CAR-T therapy on patients with CEA-positive CRC. The results revealed some efficacy in treated patients, with satisfactory tolerance of CEA CAR-T cells even at high dosages (105). CEA CAR-T cells were constructed as follows: Peripheral blood mononuclear cells were isolated from patients’ peripheral blood and then activated with immobilized CD3 and CD28 antibodies. On day 2, a polybrene-treated lentiviral vector (1×10^6^ cells/well, MOI 5) was used to infect T cells cultured with IL-2 for 12–14 days after viral transduction to determine the necessary cell dosage (105). CEA CAR-T cells, in combination with IL-12, exhibited increased antitumor activity in colorectal, pancreatic, and gastric cell lines (118). Further phase I clinical trials are underway in China (NCT05240950).

#### 3.1.4 MUC-1 and CD133

A series of trials targeting *MUC-1* and *CD133* has been performed, but further assessments of their safety and feasibility among patients with CRC are needed. MUC-1 is overexpressed in CRC and other cancer tissues and enhances neoplastic transformation and metastasis in patients with CRC (119). CD133 is a 120-kDa transmembrane glycoprotein that is expressed in hematopoietic stem and progenitor cells and localizes to membrane protrusions (120). It has been detected in many solid tumors, including colon cancer (121, 122), and its overexpression is linked to higher-stage tumors, signifying poor prognosis for most patients (123, 124). In a phase I/II trial (NCT02541370) on CAR-T immunotherapy, CRC-manipulated CD133 was used as an antigen. CART-133 cells were generated and transduced in two steps—production of lentivirus and creation of CART-133 cells. Starting with lentiviral vector generation, CAR.133 contained an anti-CD133 scFv stemming from the gene bank HW35041.1, human CD137, and CD3z signaling domains. The pseudotyped, clinical-grade lentiviral vector was generated according to the standard transient transfection protocol established by McGinley et al. (125). CART-133 cells were obtained as follows (126–129): Peripheral blood mononuclear cells were extracted and directly suspended in a medium containing an anti-CD3 mAb mixed with human recombinant IL-2. On day 3 of cell culture, lentivirus-mediated CAR transduction was conducted in six-well plates coated with a recombinant fibronectin fragment. The cells proliferated ex vivo after transduction, and IL-2 was added thrice weekly until the desired cell dose was reached. CART-133 cells have undergone phase I and II clinical trials and shown a good response with tolerable toxicity (NCT02541370) (103, 104).

In summary, a new era in cancer treatment is being persuaded in by the field’s constant pursuit of improvements in basic research on CAR-T immunotherapy. The variety of potential CAR-T treatment strategies for colorectal cancer (CRC) that are demonstrated in preclinical models and early-stage clinical trials highlight the plethora of research being done to determine the best targets or combinations of cutting-edge checkpoint inhibitors and monoclonal antibodies. Broadening the range of available therapy alternatives will provide CRC patients with long-lasting clinical advantages. Henceforth, collaborative effort, maintaining an ongoing research, and clinical evaluation are of great need to fully wrap our heads around the promise of CAR-T immunotherapy and inaugurate a game-changing revolution in the scope of CRC treatments.

### 3.2 T-cell receptor-engineered T-cell therapy

Another type of adoptive cell therapy is T-cell receptor therapy, which involves using genetic editing technology to modify a patient’s own T cells and introduce an antigen-specific gene sequence. This process generates an anti-tumor response by exclusively recognizing tumor antigens through the mediated action of TCR (130).

Although CAR-T cells have been extensively studied in CRC, TCR-T cell therapy preceded them. This concept dates back to 1986 when Dembić et al. successfully redefined the specificity of T cells through the transduction of MHC-restricted TCRα and TCRβ genes into mouse T cells (131) (**Figure 2A**).

TCR T cell therapy in CRC is in early stages in clinical trials due to many hurdles and setbacks noticed in few studies and its efficacies and safety are yet to be demonstrated (132). In 2011, the results of a clinical trial led by Parkhurst et al. were disclosed. The investigation focused on crafting T cells with TCRs designed to target CEA, with the aim of serving as a therapeutic intervention for three patients resistant to conventional treatments and presenting tumors with elevated CEA expression. Approximately 5-6 months post-treatment, two patients experienced disease progression, while the remaining patients exhibited no therapeutic effect, despite previous evidence of the regimen’s anti-cancer activity. Furthermore, a drawback of this regimen is that all patients experienced severe colitis, indicating the potential targeting of healthy intestinal cells by TCR T cells. Consequently, the trial was suspended; however, it showcased the practicability of TCR T cell therapy in mCRC setting while highlighting its backsides and restriction in the use of CEA as a target in this modality (133).

Consequently, few clinical trials are found in ClinicalTrials.gov website of which with suspended, terminated, recruiting, active non-recruiting, and completed. These trials with the following NCT number: NCT03970382, NCT01723306, NCT03431311, NCT03638206, NCT05124743, NCT05451849, NCT05292859, NCT06043713, NCT05194735, and NCT00496860. The application of TCR T cell therapy in treating CRC is assumed to play a crucial role against solid tumors, driven by groundbreaking technologies and advancements in the field of tumor immunology.

Finally, it’s noteworthy that in January 2022, a milestone has been achieved in this treatment modality—FDA authorization of tebentafusp, a bispecific gp100 peptide human leukocyte antigen HLA-A*02:01-directed TCR CD3 T cell engager against metastatic melanoma. Tebentafusp is a game-changer and will pave the way for extensive research to advance this therapy and overcome its challenges in solid tumors.

## 4 Immune checkpoints: biological aspects and clinical studies

As the primary effector cells in the immune response against tumors, T lymphocytes identify and mediate cytotoxicity against antigenic molecules arising from the genetic and epigenetic changes that characterize malignant transformation (134). APCs, which display antigenic peptides, are recognized by the TCR, thereby initiating the MHC-mediated immune response. Cytokine production, T-cell lysis, and effector cell response are all dependent on surplus costimulatory signals through the B7 protein (134). The B7 protein can pair with CD28 on T cells, generating an amplified TCR signal, or with CTLA-4 on T cells, suppressing T-cell activation. Throughout long-term antigen exposure, the inhibitory receptor of PD-1 is expressed by T cells, causing the suppression of T cells through interaction with PD-L1, which is expressed in the TME. Immune checkpoint blockade via mAbs leads to the preferential activation of cancer-specific T cells and revival of tumor immunity (134). As a camouflaging mechanism, tumors often activate this immune blockade to gain protection against immune surveillance; hence, ICIs can revive tumor immunity and make tumors vulnerable to immune cells (134). Because CRC is liable to evade immunosurveillance via different mechanisms (135), interest in using ICIs as cancer therapies is growing. CTLA-4, PD-1, and lymphocyte activation gene 3 (LAG-3) immune checkpoints can be classified as immunotherapeutic targets that impede cancer growth (136–138). In this section, we describe anti-CTLA-4 and anti-PD-1 agents and their clinical studies. All data were obtained from ClinicalTrials.gov, and only ClinicalTrials.gov-registered trials were thus included.

### 4.1 Exploring potential immune checkpoint targets for CRC: an analytical overview of counteracting mechanisms

PD1 (CD279) is found on the cell surface of T lymphocytes CD8+ and CD4+, natural killer cells (NK), B lymphocytes, and tumor-infiltrating lymphocytes (TILs) (139). It has a crucial function in maintaining the equilibrium of tumor immunity and inflammatory responses, thereby reducing the immune response caused by T lymphocytes that have traveled to the tumor microenvironment. In normal tissues, this mechanism serves to prevent prolonged and repetitive tissue injury that can lead to permanent damage. There are two ligands that PD1 interacts with (140). One of them is PD-L1, which is found on the surface of activated lymphocytes, peripheral tissues and organs, and tumor cells. The other ligand is PD-L2, which is primarily expressed by macrophages and dendritic cells (140). When T cells become exhausted, they lose their ability to carry out their effector function. This is indicated by the expression of PD1 (139). The interaction between PD1 and PD-L1/2 inhibits T cell activation and the secretion of cytokines such as interferon-γ (IFN-γ), tumor necrosis factor-α (TNF-α), and interleukin 2 (IL-2) (139). This interaction plays a role in maintaining immune homeostasis and preventing the development of autoimmunity (141).

Inhibition of the PD-1/PD-L1 pathway through the administration of monoclonal antibodies (mAbs) has the potential to reactivate the function of cytotoxic T lymphocytes (CTLs) and their capacity to attack tumor cells (139). This pathway has been identified as a negative modulator of immune response, as it restricts the function of TILs in the tumor immune microenvironment (TIME) (139).

Immunoregulatory cells and immune mediators have the potential to modulate T cells activity (142–144). In the tumor microenvironment, tumor cells dysregulate the expression of immune-checkpoint inhibitors to favor the immune resistance process and exhaust/diminish cytotoxic T cells activity leading to tumor survival and growth (145). T cells encode for CTLA-4 protein that controls immune reactions. CTLA-4 prevents T cells from destroying other cells, particularly cancer cells, when associated with the B7 protein (146). CTLA-4 is among the among the blockade immune checkpoint inhibitors used. The immune system’s “brakes” are released, and the capacity of T cells to eradicate cancer cells increases when this protein is suppressed (140). By blocking inhibitory immunological checkpoints, CTLs may prevent CRC proliferation and increase the immune response to malignancy (140). The use of monoclonal antibodies (mAbs) to inhibit CTLA-4 action is an encouraging anticancer approach that enhances T cell activation and increases antitumor activity (147). Anti-CTLA-4 antibodies will bind to CTLA-4/B7 receptors on the surface of T cells, thereby extending T cell activity and enhancing their potential (118). The suppressive element of the immune system, Treg cells, constitutively expresses CTLA-4; thus, utilizing anti-CTLA-4 mAbs may augment antitumor action by suppressing the Treg cell function (119).

Apart from CTLA-4 and PD-1, LAG-3 (or CD223) and T-cell Ig- and mucin domain-3-containing molecule 3 (TIM-3) are expressed on activated and dysfunctional T cells. LAG-3 has various biological effects on T-cell function. It negatively affects the activation, proliferation, and homeostasis of T cells and has been implicated in the suppressive function of Tregs (148–150).With PD-1, LAG-3 sustains CD8+ T-cell exhaustion during chronic viral infections (151) and helps maintain CD8+ T cells in a tolerogenic state (152).

### 4.2 ICIs in clinical trials

Numerous pharmacological and biochemical investigations have revealed that signaling molecules play a role in CRC development and spread both *in vitro* and *in vivo*. Epidermal growth factor, vascular endothelial growth factor, and hepatocyte growth factor and its cognate ligand are involved in this relationship as emerging targets for mAb therapy for CRC (69).

The FDA has approved several mAbs for CRC treatment, including cetuximab, bevacizumab, panitumumab, ramucirumab, ipilimumab, and pembrolizumab, which have demonstrated a good response for cancer remission. More antibody therapy trials are being conducted, with avelumab being tested in phase III trials and rilotumumab, trastuzumab, pertuzumab, tremelimumab, nivolumab, camrelizumab, atezolizumab, and durvalumab in phase II trials. These studies preliminarily demonstrate the protective effect of specific mAbs against weak CRC cells (69, 73). **Table 3** and **Supplementary Table 1** display ongoing and completed clinical trials involving ICIs for CRC.

**Table 3:** Ongoing clinical trials using ICI in CRC.

Intervention Treatment	Trial Title	Phase	Actual Enrollment (# of participants)	NCT Number	Ref
Pembrolizumab Radiotherapy Radiofrequency ablation	Assess the Efficacy of Pembrolizumab Plus Radiotherapy or Ablation in Metastatic Colorectal Cancer Patients	Phase 2	34	NCT02437071	clinicaltrials.gov
Pembrolizumab/Regorafenib	Regorafenib and Pembrolizumab in Treating Participants With Advanced or Metastatic Colorectal Cancer	Phase 1 Phase 2	75	NCT03657641	clinicaltrials.gov
Favezelimab pembrolizumab/ regorafenib/TAS-102	A Study of Coformulated Favezelimab/Pembrolizumab (MK-4280A) Versus Standard of Care in Subjects With Previously Treated Metastatic PD-L1 Positive Colorectal Cancer (MK-4280A-007) Colorectal	Phase 3	432	NCT05064059	clinicaltrials.gov
Pembrolizumab/ Pemetrexed/Oxaliplatin/Dexamethason Dietary Supplement: Folic Acid/: Vitamin B-12	Study of Pembrolizumab With Pemetrexed and Oxaliplatin in Chemo-Refractory Metastatic Colorectal Cancer Patients	Phase 1	33	NCT03626922	clinicaltrials.gov
grapiprant and pembrolizumab	Grapiprant and Pembrolizumab in Patients With Advanced or Progressive MSS Colorectal Cancer	Phase 1	54	NCT03658772	clinicaltrials.gov
Biological: Pembrolizumab Drug: Binimetinib/ Oxaliplatin/ Leucovorin/5-Fluorouracil [5-FU]/Irinotecan	Safety and Efficacy of Pembrolizumab (MK-3475) Plus Binimetinib Alone or Pembrolizumab Plus Chemotherapy With or Without Binimetinib in Metastatic Colorectal Cancer (mCRC) Participants (MK-3475-651)	Phase 1	220	NCT03374254	clinicaltrials.gov
Pembrolizumab /Bevacizumab/Binimetinib	Study of Pembrolizumab, Binimetinib, and Bevacizumab in Patients With Refractory Colorectal Cancer	Phase 2	53	NCT03475004	clinicaltrials.gov
Stereotactic body radiotherapy (SBRT)/Pembrolizumab	PI Pembro in Combination With Stereotactic Body Radiotherapy for Liver Metastatic Colorectal Cancer	Phase 1	18	NCT02837263	clinicaltrials.gov
Entinostat Pembrolizumab	Ph1b/2 Dose-Escalation Study of Entinostat With Pembrolizumab in NSCLC With Expansion Cohorts in NSCLC, Melanoma, and Colorectal Cancer	Phase 1 Phase 2	202	NCT02437136	clinicaltrials.gov
Pembrolizumab combined with other drugs	Study of Pembrolizumab (MK-3475) vs Standard Therapy in Participants With Microsatellite Instability-High (MSI-H) or Mismatch Repair Deficient (dMMR) Stage IV Colorectal Carcinoma (MK-3475-177/KEYNOTE-177	Phase 3	307	NCT02563002	clinicaltrials.gov
Bevacizumab/ Capecitabin/ Pembrolizumab Other: Laboratory Biomarker Analysis	Pembrolizumab, Capecitabine, and Bevacizumab in Treating Patients With Microsatellite Stable Colorectal Cancer That Is Locally Advanced, Metastatic, or Cannot Be Removed by Surgery	Phase 2	44	NCT03396926	clinicaltrials.gov
pembrolizumablenvatinib regorafenib TAS-102 (trifluridine and tipiracil)	Study of Lenvatinib (MK-7902/E7080) in Combination With Pembrolizumab (MK-3475) Versus Standard of Care in Participants With Metastatic Colorectal Cancer (MK-7902-017/E7080-G000-325/LEAP-017)	Phase 3	434	NCT04776148	clinicaltrials.gov
TEW-7197	Vactosertib in Combination With Pembrolizumab in Metastatic Colorectal or Gastric Cancer	Phase 1 Phase 2	67	NCT03724851	clinicaltrials.gov
Regorafenib/Nivolumab/Pembrolizumab/ Camrelizumab/Sintilimab/ Toripalimab/ Tislelizumab	Regorafenib Plus Programmed Cell Death-1 (PD-1) Inhibitors in Patients With Advanced Colorectal Cancer		100	NCT04771715	clinicaltrials.gov
galinpepimut-S Pembrolizumab	Galinpepimut-S in Combination With Pembrolizumab in Patients With Selected Advanced Cancers	Phase 1 Phase 2	90	NCT03761914	clinicaltrials.gov
XL888 Pembrolizumab	Pembrolizumab and XL888 in Patients With Advanced Gastrointestinal Cancer	Phase 1	49	NCT0309578	N clinicaltrials.gov
Pembrolizumab Combined with other ICIs and other treatment	Predictive Value of Drug Sensitivity Testing Tumorspheres From Patients With Metastatic Colorectal Cancer	Phase 2	90	NCT0325161	clinicaltrials.gov
Pembrolizumab Ziv-Aflibercept	Testing the PD-1 Antibody, MK3475, Given With Ziv-aflibercept in Patients With Advanced Cancer	Phase 1	78	NCT02298959	clinicaltrials.gov
THOR-707/Pembrolizumab/Cetuximab	A Study of SAR444245 Combined With Other Anticancer Therapies for the Treatment of Participants With Gastrointestinal Cancer (Master Protocol) (Pegathor Gastrointestinal 203)	Phase 2	280	NCT05104567	clinicaltrials.gov
Pembrolizumab and Lenvatinib	Efficacy and Safety of Pembrolizumab (MK-3475) Plus Lenvatinib (E7080/MK-7902) in Previously Treated Participants With Select Solid Tumors (MK-7902-005/E7080-G000-224/LEAP-005)	Phase 2	590	NCT03797326	clinicaltrials.gov
CPI-006/ ciforadenanto/pembrolizumab	CPI-006 Alone and in Combination With Ciforadenant and With Pembrolizumab for Patients With Advanced Cancers	Phase 1	378	NCT03454451	clinicaltrials.gov
Pembrolizumab/Trebananib	Pembrolizumab (Anti-PD-1) and AMG386 (Angiopoietin-2 (Ang-2) in Patients With Advanced Solid Tumor	Phase 1	60	NCT03239145	clinicaltrials.gov
ONCR-177/pembrolizumab	Study of ONCR-177 Alone and in Combination With PD-1 Blockade in Adult Subjects With Advanced and/or Refractory Cutaneous, Subcutaneous or Metastatic Nodal Solid Tumors or With Liver Metastases of Solid Tumors	Phase 1	132	NCT04348916	clinicaltrials.gov
Pembrolizumab Combined with other ICIs and other treatments	QUILT-3.055: A Study of Combination Immunotherapies in Patients Who Have Previously Received Treatment With Immune Checkpoint Inhibitors	Phase 2	145	NCT03228667	clinicaltrials.gov
ASP1951/pembrolizumab	A Study of ASP1951 in Subjects With Advanced Solid Tumors	Phase 1	120	NCT03799003	clinicaltrials.gov
Pembrolizumab Combined with other ICIs and other treatments	A Phase 1 Study of Pegilodecakin (LY3500518) in Participants With Advanced Solid Tumors	Phase 1	350	NCT02009449	clinicaltrials.gov
SBT6050/pembrolizumab/Cemiplimab	A Study of SBT6050 Alone and in Combination With PD-1 Inhibitors in Subjects With Advanced HER2 Expressing Solid Tumors	Phase 1	58	NCT04460456	clinicaltrials.gov
Pembrolizumab and Combined with other ICIs and other product	TTX-030 in Combination With Immunotherapy and/or Chemotherapy in Subjects With Advanced Cancers	Phase 1	185	NCT04306900	clinicaltrials.gov
Nivolumab	A Phase II Trial Assessing Nivolumab in Class II Expressing Microsatellite Stable Colorectal Cancer (ANICCA)	Phase 2	35	NCT03981146	clinicaltrials.gov
Regorafenib Nivolumab	Regorafenib and Nivolumab in Mismatch Repair (MMR) Refractory Colorectal Cancer	Phase 1	52	NCT03712943	clinicaltrials.gov
Ipilimumab Nivolumab Radiation Therapy	Nivolumab+Ipilimumab+RT in MSS mCRC	Phase 2	32	NCT04575922	clinicaltrials.gov
Drug: Nivolumab Drug: FLOX	METIMMOX: Colorectal Cancer METastasis - Shaping Anti-tumor IMMunity by OXaliplatin (METIMMOX)	Phase 2	80	NCT03388190	clinicaltrials.gov
Drug: Metformin Biological: Nivolumab	Nivolumab and Metformin in Patients With Treatment Refractory MSS Colorectal Cancer	Phase 2	24	NCT03800602	clinicaltrials.gov
Ipilimumab Nivolumab Regorafenib	Regorafenib, Ipilimumab and Nivolumab for the Treatment of Chemotherapy Resistant Microsatellite Stable Metastatic Colorectal Cancer	Phase 1	39	NCT04362839	clinicaltrials.gov
Copanlisib Nivolumab	Study of PI3Kinase Inhibition (Copanlisib) and Anti-PD-1 Antibody Nivolumab in Relapsed/Refractory Solid Tumors With Expansions in Mismatch-repair Proficient (MSS) Colorectal Cancer	Phase 1 Phase 2	54	NCT03711058	clinicaltrials.gov
Anti-SEMA4D Monoclonal Antibody VX15/2503 Ipilimumab Nivolumab	VX15/2503 and Immunotherapy in Resectable Pancreatic and Colorectal Cancer	Phase 1	10	NCT03373188	clinicaltrials.gov
Nivolumab Immunotherapy Relatlimab	iSCORE: Immunotherapy Sequencing in COlon and REctal Cancer	Phase 2	25	NCT03867799	clinicaltrials.gov
Ipilimumab 200 MG in 40 ML Injection Nivolumab 10 MG/ML	Interest of iRECIST Evaluation for DCR for Evaluation of Patients With Deficient MMR and /or MSI Metastatic Colorectal Cancer Treated With Nivolumab and Ipilimumab (NIPICOL)	Phase 2	57	NCT03350126	clinicaltrials.gov
Drug: Ipilimumab Drug: Nivolumab Drug: Cobimetinib Drug: Daratumumab Drug: BMS-986016	A Study of Nivolumab Alone or Nivolumab Combination Therapy in Colon Cancer That Has Come Back or Has Spread (CheckMate142)	Phase 2	385	NCT02060188	clinicaltrials.gov
Biological: VE800 Drug: Nivolumab Drug: Vancomycin Oral Capsule	Study of VE800 and Nivolumab in Patients With Selected Types of Advanced or Metastatic Cancer (Consortium-IO)	Phase 1 Phase 2	54	NCT04208958	clinicaltrials.gov
TP-1454 monotherapy TP-1454 combination therapy in combination with ipilimumab and nivolumab	Phase 1 Study of Oral TP-1454	Phase 1	44	NCT04328740	clinicaltrials.gov
Drug: mFOLFOX6 Biological: MVA-BN-CV301 Biological: FPV-CV301 Drug: Nivolumab	A Trial of Perioperative CV301 Vaccination in Combination With Nivolumab and Systemic Chemotherapy for Metastatic CRC	Phase 2	78	NCT03547999	clinicaltrials.gov
Nivolumab and large number of drugs (chosen according to genetic testing)	Targeted Therapy Directed by Genetic Testing in Treating Patients With Advanced Refractory Solid Tumors, Lymphomas, or Multiple Myeloma (The MATCH Screening Trial)	Phase 2	6452	NCT02465060	clinicaltrials.gov
Nivolumab combined with other ICIs and other treatments	A Phase 1 Study of Pegilodecakin (LY3500518) in Participants With Advanced Solid Tumors (IVY)	Phase 1	350	NCT02009449	clinicaltrials.gov
Nivolumab	A Phase II Trial Assessing Nivolumab in Class II Expressing Microsatellite Stable Colorectal Cancer (ANICCA)	Phase 2	35	NCT03981146	clinicaltrials.gov
Regorafenib Nivolumab	Regorafenib and Nivolumab in Mismatch Repair (MMR) Refractory Colorectal Cancer	Phase 1	52	NCT03712943	clinicaltrials.gov
Ipilimumab Nivolumab Radiation Therapy	Nivolumab+Ipilimumab+RT in MSS mCRC	Phase 2	32	NCT04575922	clinicaltrials.gov
Drug: Nivolumab Drug: FLOX	METIMMOX: Colorectal Cancer METastasis - Shaping Anti-tumor IMMunity by OXaliplatin (METIMMOX)	Phase 2	80	NCT03388190	clinicaltrials.gov
Drug: Metformin Biological: Nivolumab	Nivolumab and Metformin in Patients With Treatment Refractory MSS Colorectal Cancer	Phase 2	24	NCT03800602	clinicaltrials.gov
Ipilimumab Nivolumab Regorafenib	Regorafenib, Ipilimumab and Nivolumab for the Treatment of Chemotherapy Resistant Microsatellite Stable Metastatic Colorectal Cancer	Phase 1	39	NCT04362839	clinicaltrials.gov
Copanlisib Nivolumab	Study of PI3Kinase Inhibition (Copanlisib) and Anti-PD-1 Antibody Nivolumab in Relapsed/Refractory Solid Tumors With Expansions in Mismatch-repair Proficient (MSS) Colorectal Cancer	Phase 1 Phase 2	54	NCT03711058	clinicaltrials.gov
Atezolizumab Bevacizumab Capecitabine	Capecitabine and Bevacizumab With or Without Atezolizumab in Treating Patients With Refractory Metastatic Colorectal Cancer	Phase 2	133	NCT02873195	clinicaltrials.gov
Obinutuzumab Atezolizumab Cibisatamab Tocilizumab	A Phase Ib Study to Evaluate the Safety, Efficacy, and Pharmacokinetics of Cibisatamab in Combination With Atezolizumab After Pretreatment With Obinutuzumab in Participants With Previously Treated Metastatic Colorectal Adenocarcinoma	Phase 1	47	NCT03866239	clinicaltrials.gov
Anti-PD-L1/TGFbetaRII Fusion Protein M7824	M7824 in Patients With Metastatic Colorectal Cancer or With Advanced Solid Tumors With Microsatellite Instability	Phase 1 Phase 2	74	NCT03436563	clinicaltrials.gov
Cabozantinib atezolizumab	Study of Cabozantinib in Combination With Atezolizumab to Subjects With Locally Advanced or Metastatic Solid Tumors	Phase 1 Phase 2	1732	NCT03170960	clinicaltrials.gov
Durvalumab	Durvalumab for MSI-H or POLE Mutated Metastatic Colorectal Cancer	Phase 2	33	NCT03435107	clinicaltrials.gov
Danvatirsen Durvalumab	Danvatirsen and Durvalumab in Treating Patients With Advanced and Refractory Pancreatic, Non-Small Cell Lung Cancer, and Mismatch Repair Deficient Colorectal Cancer	Phase 2	53	NCT02983578	clinicaltrials.gov
Durvalumab Olaparib Cediranib	Basket Combination Study of Inhibitors of DNA Damage Response, Angiogenesis and Programmed Death Ligand 1 in Patients With Advanced Solid Tumors	Phase 2	90	NCT03851614	clinicaltrials.gov
Durvalumab Radiation Therapy Tremelimumab	Durvalumab and Tremelimumab With or Without High or Low-Dose Radiation Therapy in Treating Patients With Metastatic Colorectal or Non-small Cell Lung Cancer	Phase 2	180	NCT02888743	clinicaltrials.gov
Combination of Durvalumab With other products	Naptumomab Estafenatox in Combination With Durvalumab in Subjects With Selected Advanced or Metastatic Solid Tumors	Phase 1	60	NCT03983954	clinicaltrials.gov
IBI310 (anti-CTLA-4 antibody) Sintilimab (anti-PD-1 antibody)	IBI310 in Combination With Sintilimab in Patients With DNA Mismatch Repair Deficient (dMMR)/Microsatellite Instability High (MSI-H) Locally-advanced or Metastatic Colorectal Cancer	Phase 2	4	NCT04258111	clinicaltrials.gov
Drug: Tremelimumab (anti-CTLA-4) Drug: Durvalumab (anti-PD-L1)	Basket Trial for Combination Therapy With Durvalumab (Anti-PDL1) (MEDI4736) and Tremelimumab (Anti-CTLA4) in Patients With Metastatic Solid Tumors (MATILDA)	Phase 2	88	NCT03982173	clinicaltrials.gov
FOLFOX regimen FOLFIRI Protocol Avelumab Panitumumab Cetuximab Bevacizumab Aflibercept	Standard Chemotherapy vs Immunotherapie in 2nd Line Treatment of MSI Colorectal Mestastatic Cancer (SAMCO)	Phase 2	132	NCT03186326	clinicaltrials.gov
Biological: Lorigerlimab	MGD019 DART® Protein in Unresectable/Metastatic Cancer	Phase 1	287	NCT03761017	clinicaltrials.gov

#### 4.2.1 Comprehensive insights into PD-1/PD-L1 inhibitors and emerging therapies for CRC

Pembrolizumab (Keytruda^®^, Merck) is a humanized mAb that targets PD-1. It inhibits the binding of PD-1 to its ligands (PD-L1 or PD-L2), thus enhancing the recognition of tumor cells by cytotoxic T cells. Pembrolizumab was first approved by the United States FDA in 2016 for the treatment of patients with metastatic non-small-cell lung cancer (NSCLC) whose tumors express PD‐L1 (153). In 2020, the FDA approved pembrolizumab for the treatment of patients with unresectable or microsatellite instability-high (MSI-H) mCRC with no prior systemic treatment for advanced disease (154).

Numerous completed and ongoing clinical trials have investigated the clinical effects of pembrolizumab on advanced CRC (**Table 3**; **Supplementary Table 1**). In the phase II clinical study KEYNOTE-164, the antitumor activity of pembrolizumab was assessed in patients with MSI-H mCRC. The data confirmed that pembrolizumab treatment had a clinical benefit (155). Antidrug antibodies, which interfere with target binding and reduce the efficacy of the drug, were detected in only 2.85% of treated patients (156). However, some patients showed a degree of resistance to pembrolizumab, and others failed to exhibit the desired response. One possible explanation for these undesirable outcomes is the immunosuppressive activity of infiltrating immune cells. Therefore, blocking these cells and enhancing the T-cell response will modulate the TME from cold to hot; this can be achieved by using anti-PD-1 drugs in combination with other immunotherapeutic agents.

Herting et al. showed that combining pembrolizumab with standard FOLFOX chemotherapy for the treatment of mCRC was safe but did not significantly improve the median progression-free survival (PFS) and median OS compared with chemotherapy alone. The immune response following combined chemotherapy and immune checkpoint blockade was assessed based on the Response Evaluation Criteria in Solid Tumors (RECIST) and median PFS. Notably, a low TNF-α level was associated with a better RECIST score, but increased Flt3 ligand and TGF-α levels were associated with an improved median PFS. Furthermore, immune checkpoint receptors on CD4+ and CD8+ T cells were compared with the RECIST response. Patients with a low expression of PD-1 and CD4+ checkpoint molecules BTLA or LAG-3 on T cells at baseline had a better RECIST CD8+ response (157). Other studies combined pembrolizumab with the GVAX colon vaccine, a GM-CSF-secreting cellular immunotherapy that induces T-cell immunity against a broad range of colon cancer-associated antigens, aiming to change the TME and induce tumor-infiltrating lymphocytes in sensitive cancers. Although no difference in objective responses was observed, a significant decrease in tumor marker levels was detected (158). Another approach for TME modulation—use of the CCR5 inhibitor maraviroc in combination with pembrolizumab—was investigated in a previous study. CCR5 is a potent regulator of the recruitment of immunosuppressive M2 macrophages, supporting tumor growth and angiogenesis. The study showed that antitumor chemokines surged during treatment, including eotaxin, which was linked to OS (159). Similar strategies based on blocking tumor-associated macrophages similar to the M2 immunosuppressive phenotype by blocking colony-stimulating factor 1 receptor using AMG 820, an antibody directed against human colony-stimulating factor 1 receptor, in combination with pembrolizumab showed preliminary evidence of activity [clinical benefit rate (irPR and irSD) of 36%] (160). A detailed analytical study of macrophage-targeted immunotherapies used a CXCL12 inhibitor, NOX-A12 (olaptesed pegol), which inhibits the binding of CXCL12 to both CXCR4 and CXCR7 receptors, showed a reduction in the number of CD14+CD15+ cells in the anti-CXCL12-treated group and in the number of CD11b+ cells in the biopsies of treated patients. The treatment was well tolerated, and long-term disease stabilization was achieved, with a disease control rate of 25%. A reasonable interpretation of NOX-A12-mediated modulation in the TME is based on the alteration of cytokine status that favors a good inflammatory cell profile. In this study, patients were divided into tissue responders (patients showing increased IL-2, IFN-γ, and IL-16 levels) and non-responders (patients showing reduced IL-2, IL-16, and CXCL10 levels). Interestingly, the responders showed a higher number of activated infiltrating CD3+ T cells that promote a hot TME (161). Another study investigated the use of the DNA methyltransferase inhibitor azacytidine alongside pembrolizumab and noted an increased number of CD8^+^ tumor-infiltrating lymphocytes in mCRC compared with that in the pre-treated condition (162).

Avelumab is a human anti-PD-L1 antibody that blocks the binding of PD-1 receptors and B7-1 on T cells. It also stimulates antibody-dependent cell-mediated cytotoxicity via engineered Fc gamma receptor 1 (163). Completed clinical trials of avelumab are presented in **Supplementary Table 1**. Avelumab has been approved by the FDA for the treatment of metastatic Merkel cell carcinoma and locally advanced or metastatic urothelial carcinoma. An association was observed between immune-related AEs and improved survival in patients treated with avelumab (164). The safety of combination therapy with autologous DCs and avelumab was assessed in patients with mCRC, revealing that the regimen was well tolerated, with a PFS of 3.1 months and OS of 12.2 months (165). Avelumab showed promising clinical efficacy and satisfactory survival outcomes in patients with NSCLC (166), thymoma (167), GC/gastroesophageal cancer (168), ovarian cancer (169), melanoma (170), and thyroid cancer (171).

Nivolumab is a potent ICI that targets the PD-1 receptor expressed on activated T cells. This human monoclonal anti-PD-1 immunoglobulin (Ig) G4 antibody binds its receptor with high affinity, effectively blocking the interaction between PD-1 receptors on T cells and their ligands (PD-L1 and PD-L2) on tumor cells. By inhibiting this interaction, nivolumab restores T-cell activity, releasing the brakes on the immune system and promoting antitumor immune responses (172). This mechanism has been proven to be effective in treating various types of solid tumors, including mismatch repair-deficient (dMMR) or MSI-H mCRC. Nivolumab gained FDA approval in 2014 for the treatment of advanced melanoma. Since then, it has been approved for use in various other cancer types, such as NSCLC, renal cell carcinoma, Hodgkin’s lymphoma (173), and CRC (174).

Ongoing studies are exploring nivolumab as a monotherapy or in combination with other agents for CRC, particularly advanced or metastatic cases (**Table 3**). Completed clinical trials of nivolumab are presented in **Supplementary Table 1**. Notably, trials have focused on specific patient populations, such as those with dMMR or MSI-H CRC, among whom nivolumab has demonstrated significant clinical activity. The FDA granted accelerated approval to nivolumab in July 2017 for the second-line treatment of MSI-H/dMMR CRC based on compelling data from phase II clinical studies (175). The phase II trial CheckMate142 (NCT02060188) tested the efficacy of nivolumab in patients with dMMR/MSI-H mCRC. The noted safety of nivolumab is in line with that reported in studies of other solid tumors, and no new safety concerns were noted (176). Based on these data, nivolumab was approved by the FDA for the treatment of dMMR/MSI-H mCRC in adults or children older than 12 years. In parallel, the FDA granted accelerated approval to the combination of nivolumab and ipilimumab for treating refractory MSI-H/dMMR CRC following the CheckMate142 study, whose data implied that combined ICIs could clinically benefit patients with dMMR/MSI-H mCRC (177).

The development of nivolumab has transformed the treatment landscape of multiple cancers, including dMMR/MSI-H CRC, leading to improved patient outcomes and prolonged survival. However, not all cases of dMMR/MSI-H CRC respond to immunotherapy, and primary resistance occurs in approximately 50% of patients with this subtype, suggesting significant molecular heterogeneity among dMMR/MSI-H CRC cases (178). Some CRC subtypes are less sensitive to current immunotherapies and exhibit a limited response to single-agent ICIs. Therefore, the key challenge is to modify CRC subtypes into highly immunogenic tumors similar to MSI-H CRC, which is sensitive to immunotherapy. Continued research and clinical trials are necessary to fully unlock the potential of nivolumab in CRC treatment.

Budigalimab (ABBV-181) is an innovative and promising mAb also designed to target PD-1. It is a humanized recombinant antibody that consists of the complementarity-determining regions of a murine antibody grafted onto frameworks of human IgG1 heavy and kappa light chains. It has been modified to effectively target PD-1 while minimizing interaction with FcγRs and reducing its FcγR-mediated effector function (179). This ICI is currently under investigation and has not yet been granted FDA approval. However, its potent PD-1-blocking activity and high specificity have shown promise in preclinical and early clinical studies, generating interest in its potential therapeutic applications. Ongoing clinical trials are exploring its efficacy and safety as a monotherapy or in combination with other agents, such as chemotherapy or targeted therapies, in patients with CRC (NCT04306900). The first-in-human study of budigalimab demonstrated that it was well tolerated and safe and exhibited an efficacy comparable to that of other PD-1 inhibitors approved for clinical use (180, 181), suggesting that it could be a promising new treatment option for patients with CRC. While clinical trials of budigalimab for CRC are ongoing, several important discussion points have arisen, including the identification of predictive biomarkers to guide patient selection, optimal combination strategies to enhance its efficacy, and potential for resistance development. Continued research and clinical investigations are crucial to fully elucidate the potential of budigalimab in CRC treatment.

Tislelizumab (BGB-A317), another anti-PD-1 monoclonal IgG4 antibody, is an emerging ICI that has shown great potential for cancer treatment. Developed by BeiGene, this humanized mAb binds PD-1 with high affinity, leading to potent T-cell activation and antitumor immune responses. The structure of tislelizumab has been modified to maximize its ability to inhibit PD-1/PD-L1 interactions and minimize its binding to FcγR, which is a potential mechanism of resistance to anti-PD-1 therapy (182). Tislelizumab was designed to specifically minimize FcγR binding on macrophages to limit antibody-dependent cellular phagocytosis (183). This unique feature of tislelizumab makes it an exciting new addition to the arsenal of currently available cancer therapies. Tislelizumab received FDA approval for the treatment of esophageal cancer, hepatocellular carcinoma in 2019, and GC/gastroesophageal cancer in 2020. Its efficacy has also been investigated in other malignant tumors, such as nasopharyngeal carcinoma, esophageal squamous cell carcinoma, CRC, and MSI-H or dMMR tumors, with acceptable adverse effects.

Ongoing clinical trials are evaluating the potential of tislelizumab in advanced CRC treatment. These clinical trials aim to assess the efficacy, safety, and long-term outcomes of tislelizumab in patients with CRC. A phase II study (NCT03469557) revealed that tislelizumab’s positive results contributed to its orphan designation by the FDA for the treatment of GC/gastroesophageal cancer, while a phase III study (NCT03777657) confirmed the potential of adding tislelizumab to chemotherapy. While tislelizumab is not yet approved by the FDA for CRC treatment, a phase II study in China (NCT03736889) showed satisfactory antitumor effects, leading to the acceptance of its listing application by the National Medical Product Administration (182).

Dostarlimab (JEMPERLI) is another humanized anti-PD-1 IgG4-isotype mAb that inhibits PD-1 interaction with both PD-L1 and PD-L2. This immune checkpoint blockade strategy enables the immune system to recognize and eliminate tumor cells without being suppressed by the TME (184). Being an IgG4 isotype therapeutic antibody, it has a substantially low need for Fc activity, making it suitable for use as a functional antagonist. Moreover, dostarlimab improves Teff activities *in vitro* by increasing cytokine generation (185). In April 2021, based on early clinical evidence of its efficacy and safety, the FDA granted accelerated approval to dostarlimab-gxly for adult patients with dMMR recurrent or advanced endometrial cancer that has progressed on or following a prior platinum-containing regimen (186). Further confirmational studies were conducted, and on February 9, 2023, full approval was granted for the same group of patients who are not candidates for curative surgery or radiation. Dostarlimab has demonstrated effectiveness in several cancers, including dMMR pan malignancies, second-line dMMR endometrial cancer, and NSCLC. Remarkably, a breakthrough clinical trial (NCT04165772) reported a 100% remission rate for rectal cancer in June 2022, providing evidence that tumor genetics can be matched with the appropriate therapy to yield a marked response. The trial is ongoing and enrolling patients with gastric, prostate, and pancreatic cancers. Dostarlimab is currently recommended for rectal cancer, and further clinical trials are exploring its potential in the treatment of various other cancers, including CRC. These trials aim to identify new therapeutic options for patients with limited treatment options and gain insights into the potential of immunotherapeutic approaches for the treatment of advanced solid tumors.

Two phase I trials are combining dostarlimab with other mAbs that enhance either T-cell function and PD-1 blockade activity (TSR-033; NCT03250832, 2017) or antitumor responses and immune-mediated tumor cell killing (GSK4381562; NCT05277051, 2022). Moreover, a single-arm phase II trial (NCT05239546, 2023) is investigating the neoadjuvant use of dostarlimab in patients with stage II and III dMMR colon cancers with the goal of avoiding surgical resection. The future of cancer treatment lies in a personalized approach that considers the cancer type and subtype. The promising effects of dostarlimab among patients with rectal cancer give hope that similarly effective treatments can be found for other cancers. However, safety studies are still necessary to identify higher-risk categories, and access to medical teams that can monitor patients and intervene if tumors recur is crucial. Overall, dostarlimab is a promising immunotherapeutic agent for the treatment of several cancer types, and ongoing clinical trials will further improve the understanding of its potential benefits.

Atezolizumab, another high-affinity humanized IgG1 antibody against PD-L1, is approved by the FDA for the treatment of metastatic NSCLC after platinum-containing chemotherapy failure. Active and completed clinical trials of atezolizumab are presented in **Table 3** and **Supplementary Table 1**. The drug was first investigated in a phase I study including patients with non-curable advanced NSCLC, melanoma, GC, renal cell carcinoma, head and neck squamous cell carcinoma, and CRC (187). Several ongoing trials are investigating the outcomes of atezolizumab use in patients with CRC. A current randomized clinical trial is also evaluating the efficacy of atezolizumab in combination with capecitabine and bevacizumab. The combination therapy showed substantially limited clinical benefits. The dual inhibition of vascular endothelial growth factor with the PD-1 or programmed death ligand 1 pathways was found to be more beneficial for patients with microsatellite-stable (MSS) and MMR-proficient (pMMR) tumors as well as for those without liver metastasis (188). When atezolizumab was combined with FOLFOXIRI/bev, patients with mCRC had a longer PFS. While there is evidence of effectiveness in patients with pMMR tumors, the benefits are noticeably more significant in patients with dMMR malignancies. Translational investigations to identify prognostic biomarkers are currently being conducted (189). In addition, the combination of cabozantinib and atezolizumab exhibited promising antitumor activity in individuals with metastatic castration-resistant prostate cancer following novel hormonal therapy while maintaining an acceptable safety profile. These findings suggest that further assessment of the combination therapy is required (190). The application of consensus molecular subtyping in the context of CRC can significantly alter the current understanding of the CRC treatment domain. In another study, an assay was devised and authenticated for use on formalin-fixed and paraffin-embedded CRC samples. The assay was subsequently introduced into a Clinical Laboratory Improvement Amendments-certified laboratory (191).

The completed clinical trial IMblaze370 showed that combination treatments with atezolizumab/cobimetinib or atezolizumab/regorafenib did not improve patients’ OS. The safety profile of the combination of atezolizumab and cobimetinib was comparable to that of the two drugs taken separately. These findings highlight the difficulty of increasing the benefits of immunotherapy for patients whose tumors have lower baseline levels of immune inflammation (192). Other initial positive outcomes of the ongoing experiment indicate that the methodology employed may yield conclusive findings within a trial framework that is both cost-effective and accommodating to patients.

The MODUL trial is expected to significantly contribute to the ongoing development of clinical trial designs and facilitate a more personalized treatment approach for patients with mCRC, in conjunction with other biomarker-driven trials that are presently in progress (193). Overall, atezolizumab has the potential to augment the immune system’s antitumor response while impeding the proliferation and metastasis of malignant cells. The combination of chemotherapy and other drugs may yield superior outcomes in the management of mCRC.

Durvalumab is an FDA-approved drug for the treatment of several cancer types. It obtained accelerated approval from the FDA in 2017 for the treatment of locally advanced or metastatic urothelial carcinoma (194). A subsequent approval was granted in 2018, allowing its use in selected patients with locally advanced, unresectable non-small cell lung cancer (NSCLC) (195). In March 2020, another milestone was achieved as durvalumab received approval for its first-line utilization in combination with chemotherapy for individuals facing extensive-stage small cell lung cancer (ES-SCLC) (196). Its approval has been expanded to reduce the risk of NSCLC progression. Durvalumab targets the PD-1/PD-L1 pathway, thus activating the immune system to attack and kill cancer cells (197). Several ongoing clinical trials show the potential of durvalumab, which exhibits favorable clinical efficacy characterized by promising response rates and satisfactory survival outcomes in patients with mCRC who possess MSI-H/dMMR or polymerase epsilon exonuclease domain mutations. Active and completed clinical trials of durvalumab are presented in **Table 3** and **Supplementary Table 1**. The clinical response to durvalumab among patients with polymerase epsilon-mutated mCRC may be limited to those with evidence of dMMR (198). Another trial showed the safety of a combination of durvalumab and tremelimumab as a neoadjuvant prior to liver resection for CRC. The therapy was found to activate T and B cells in pMMR mCRC (199). Additional trials demonstrated the safety and tolerability of PexaVec in combination with durvalumab and tremelimumab. The combined treatment with PexaVec, durvalumab, and tremelimumab exhibited promising clinical efficacy in individuals with pMMR mCRC. However, additional investigations are required to ascertain the corresponding predictive biomarkers (37). Another study showed that the combination of bevacizumab and FOLFOX with durvalumab, a PD-L1 inhibitor, and oleclumab, an anti-CD73 mAb, modestly improved response rates but did not confer any PFS advantage over standard-of-care treatment alone (200). Moreover, a multicenter randomized phase II study assessed the potential advantages of combining durvalumab with tremelimumab as a standalone treatment or in conjunction with low-dose or hypofractionated radiation among patients with metastatic NSCLC who had previously experienced progression on programmed death ligand 1-directed therapy (201). PD-L1/CTLA-4-directed therapy could be a treatment option for certain patients. Future studies should refine predictive biomarkers in this setting.

Several completed clinical trials have assessed the use of durvalumab in patients with CRC. One phase II clinical trial investigated the efficacy of dual immune checkpoint blockade using durvalumab and tremelimumab in patients with MSS mCRC who were experiencing progression on chemotherapy. The study involved the administration of palliative hypofractionated radiotherapy (SBRT). The safety and tolerability of the combination of SBRT and dual immunotherapy were found to be in accordance with standard immunotherapy guidelines (202). Another trial examined the same ICI combination and radiotherapy with regard to inducing systemic antitumor immunity in preclinical and clinical models in patients with pMMR mCRC. Both radiotherapy and the ICI in this study failed to meet the predetermined endpoint criteria, thus rendering the regimen unsuitable for further investigation. Nevertheless, there were infrequent occurrences of systemic immune enhancement and reduction in non-irradiated lesions, which were identified as an abscopal response. The feasibility of combining durvalumab and tremelimumab along with radiation therapy as well as the manageable safety profile of this approach has been confirmed in patients with MSI-H mCRC. Additional investigations into innovative immunotherapeutic combinations and the discovery of biomarkers that can anticipate abscopal responses are necessary (203). A trial of the safety of incorporating Y90 radioembolization into durvalumab and tremelimumab treatment regimens was performed. However, this combination did not elicit tumor-specific immune responses against liver-metastasized MSS CRC (204). Additionally, the combination of trametinib and durvalumab exhibited tolerability deemed acceptable in patients with refractory MSS mCRC. The initial investigation phase failed to satisfy the effectiveness standards, thereby rendering it unsuitable for advancement to the subsequent phase.

The potential impact of the site of metastatic disease on outcomes in clinical trials involving novel immunotherapeutic combinations is a subject of interest (205). In a previous study, the safety of the combination of durvalumab and IP ONCOS-102 was confirmed, as no dose-limiting toxicities were detected. The initial analyses indicated the presence of biological and clinical efficacies (206). Finally, the clinical efficacy and pharmacodynamic effects of a combination therapy involving an oral hypomethylating agent, CC-486, and durvalumab were evaluated in immunologically cold solid tumors. However, the results did not indicate significant activity in either area. The findings of this study, which included a wealth of biomarkers, provide valuable insights for ongoing drug development attempts that utilize these agents (207). Collectively, durvalumab shows promising clinical efficacy and satisfactory survival outcomes in patients with mCRC. **Table 4** compares various types of PD-1 monoclonal antibodies, addressing the pros and cons of utilizing these ICIs.

**Table 4:** Comparison of Various PD-1/PD-L1 Monoclonal Antibodies – Mechanistic Action, Pros, and Cons.

Monoclonal Antibody	Mechanism of Action	Clinical Approvals	Advantages	Disadvantages
**Pembroluzimab (Keytruda)**	PD-1 mAb, inhibits PD-1 binding to PD-L1 or PD-L2	FDA-approved for metastatic non-small-cell lung cancer (2016) and unrespectable or MSI-H mCRC (2020).	Enhances the recognition of tumor cells by cytotoxic T cells.	Some patients show resistance and immunosuppressive activity of infiltrating immune cells.
**Avelumab**	Anti-PD-L1 antibody, blocks PD-1 receptors and B7-1 on T cells. stimulates antibody-dependent cell-mediated cytotoxicity via engineered Fc gamma receptor 1.	FDA-approved for metastatic Merkel cell carcinoma and locally advanced or metastatic urothelial carcinoma.	Promising clinical efficacy and satisfactory survival outcomes observed in various cancers (NSCLC, thymoma, GC/gastroesophageal cancer, ovarian cancer, melanoma, and thyroid cancer).	Limited data on combination therapies and specific patient populations.
**Nivolumab**	PD-1 IgG4 mAb, inhibits PD-1 interaction with PD-L1 and PD-L2.	FDA approved for various cancers, including advanced melanoma (2014), 2017 for the second-line treatment of MSI-H/dMMR CRC, NSCLC, renal cell carcinoma, Hodgkin’s lymphoma and CRC	High affinity binding, effectively blocking the interaction between PD-1 receptors on T cells and their ligands (PD-L1 and PD-L2) on tumor cells, thus restoring T-cell activity Significant clinical activity observed in specific patient populations.	Primary resistance occurs in some cases, and not all CRCs respond to immunotherapy.
**Budigalimab (ABBV-181)**	PD-1 IgG1 mAb, modified for reduced FcγR interaction	Investigational, not FDA approved	Potent PD-1-blocking activity and high specificity. well tolerated and safe with high efficacy.	Not FDA approved Ongoing clinical trials is needed to provide more insights.
**Tislelizumab (BGB-A317)**	PD-1 IgG4 mAb	FDA approved for esophageal cancer, hepatocellular carcinoma (2019), and GC/gastroesophageal cancer (2020)	High affinity to PD-1 leading to potent T-cell activation and antitumor immune responses The structure has been modified to maximize its ability to inhibit PD-1/PD-L1 interactions and minimize its binding to FcγR, which is a potential mechanism of resistance to anti-PD-1 therapy	Not yet approved by the FDA for CRC treatment
**Dostarlimab (JEMPERLI)**	PD-1 IgG4- mAb, inhibits PD-1 interaction with PD-L1 and PD-L2	FDA approved for dMMR recurrent or advanced endometrial cancer (2021)	Low need for Fc activity, making it suitable for use as a functional antagonist. Improves Teff activities in vitro by increasing cytokine generation. Demonstrated effectiveness in several cancers, including dMMR pan malignancies, second-line dMMR endometrial cancer, and NSCLC.	Limited data on efficacy and safety in CRC. Ongoing trials are needed to provide more insights.
**Atezolizumab**	Anti-PD-L1 IgG1 mAb	FDA approved for metastatic NSCLC (2016)	High-affinity against PD-L1. Potential benefit in combination with specific regimens. Has the potential to augment the immune system’s antitumor response while impeding the proliferation and metastasis of malignant cells. The combination of chemotherapy and other drugs may yield superior outcomes in the management of mCRC.	Limited benefit observed in some combination therapies. Ongoing studies are required to refine predictive biomarkers.
**Durvalumab (Imfinzi)**	Anti-PD-L1 IgG1 mAb Blocks PD-1/PD-L1 interaction	FDA approved for urothelial carcinoma (2017), NSCLC (2018), ES-SCLC (2020)	Promising response rates and satisfactory survival outcomes in patients with mCRC.	Limited clinical response and safety concerns underscore the need for ongoing research and refinement of predictive biomarker. More research is required to enhance the efficacy in certain patient groups.

In summary, the integration of these therapeutic modalities exhibits promising prospects for enhancing the overall clinical prognosis of afflicted individuals. For example, the observed clinical impact is notable when considering the administration of temozolomide as a priming agent, followed by a combination therapy involving low-dose ipilimumab and nivolumab. This proof of concept study specifically focuses on patients with (MSS) and (MGMT)-silenced (mCRC). Another notable example is TPST-1120, as a monotherapy or in conjunction with Nivolumab, has exhibited favorable tolerability and demonstrated observable advancements in terms of tumor size or activity in subjects with advanced cancers. Notably, this combination approach has yielded positive responses in individuals with late-stage renal cell carcinoma (RCC) and heavily treated cholangiocarcinoma (CCA) that were previously unresponsive to anti-PD-1 therapy. Another significant finding is that the addition of atezolizumab to the initial regimen, namely FOLFOXIRI plus bevacizumab, has demonstrated both safety and an escalation in progression-free survival (PFS) among patients with previously untreated metastatic colorectal cancer (mCRC). An enhancement in survival rates was noted among patients diagnosed with high immune score (IS) pMMR mCRC when administered with this particular combination therapy as an initial treatment approach.

In contrast, several combinations have demonstrated a lack of efficacy or failure to meet their primary objective of enhancing overall survival. For instance, the combination of varlilumab and nivolumab exhibited no significant overall response. However, it is worth noting that there was a discernible response among patients who were unresponsive to anti-PD-1 therapy. An additional illustration pertains to the utilization of Pembrolizumab in conjunction with Ibrutinib for the treatment of advanced, refractory colorectal cancers. This particular therapeutic approach demonstrated a notable deficiency in its capacity to impede tumor growth within the context of metastatic colorectal cancer (mCRC). The efficacy endpoint of the combination therapy involving durvalumab and IP ONCOS-102 was not achieved.

The potential strategy for cancer immunotherapy lies in the promising concomitant utilization of PD-1 inhibitors and monoclonal antibodies (mAbs). PD-1 inhibitors, which fall under the category of immunotherapy medications, operate through the mechanism of inhibiting the PD-1 receptor present on T cells, thereby compromising their capacity to engage in the cytotoxicity against malignant cells. In contrast, monoclonal antibodies (mAbs) are artificially engineered molecules with the purpose of selectively attaching to particular targets on cancer cells, such as receptors or proteins. This targeted binding aims to trigger an immune response against cancerous cells.

Despite these advancements, there are challenges in the widespread adoption of mAbs for colorectal cancer treatment. Issues such as patient selection, biomarker identification, and resistance mechanisms need to be addressed to optimize the use of mAbs in clinical practice.

#### 4.2.2 CTLA-4 as an immunotherapeutic target in CRC: diverse approaches and potential combinations

Ipilimumab is another ICI that has received FDA approval for the therapeutic management of melanoma and certain types of lung cancer (208). It is an anti-CTLA-4 mAb. Several ongoing clinical trials are investigating its use in combination with other drugs for treating CRC. One trial aimed to identify the recommended phase II dosage of regorafenib, ipilimumab, and nivolumab and assess their efficacy in an expanding cohort of patients with MSS mCRC. In this non-randomized clinical study, the combination showed promising therapeutic action in patients without liver metastases. Nevertheless, randomized clinical studies should be conducted to validate these findings (209). In a different study, pseudoprogression was uncommon in patients with MSI/dMMR mCRC treated with nivolumab and ipilimumab. This combined ICI treatment conferred a remarkable disease control rate and survival rate (210). The completed MAYA trial showed the benefit of temozolomide priming followed by low doses of ipilimumab and nivolumab, which resulted in long-term therapeutic benefits in patients with MSS and O^6^-methylguanine-DNA methyltransferase-silenced mCRC (211).

Another ongoing clinical trial examining XmAb^®^22841 as a monotherapy or in combination with pembrolizumab is evaluating the maximum tolerated dose and/or recommended dose of XmAb22841. The study aims to evaluate the safety, tolerability, pharmacokinetics, immunogenicity, and antitumor activity of XmAb22841 in patients with advanced solid tumors (212). XmAb22841 is a bispecific Fc-engineered antibody targeting the human negative immunoregulatory checkpoint receptors CTLA-4 and LAG-3, both members of the Ig superfamily. This antibody exhibits potential immune checkpoint inhibition and antineoplastic properties. Upon administration, XmAb22841 binds to both CTLA-4 and LAG-3 expressed on T cells within the TME. Tregs overexpress CTLA-4 and LAG-3 in the TME, inhibiting T-cell proliferation and activation. With XmAb22841 treatment, both CTLA-4 and LAG-3 checkpoint receptors are simultaneously blocked, enhancing T-cell activation and proliferation more effectively than blocking a single checkpoint receptor alone. Engineering the Fc domain can also increase the stability and half-life of antibodies. Furthermore, the safety of durvalumab and tremelimumab with or without stereotactic body radiation therapy in relapsed small-cell lung cancer has been proven (213).

A completed clinical trial using ticilimumab as a monotherapy showed no substantial effect despite the survival of 21 patients for more than 6 months and the intriguing mild response of one patient. The treatment might be promising when combined with other ICIs (214). A combination of Y90 and durvalumab or durvalumab and tremelimumab can be safe.

In another trial (NCT04258111), the effectiveness and safety of IBI310 in combination with sintilimab in patients with locally advanced or MSI-H/dMMR mCRC were evaluated. Ongoing and completed clinical trials of anti-CTLA-4 are presented in **Table 3** and **Supplementary Table 1**.

In conclusion, the efficacy of targeting anti-CTLA-4 as a monotherapy in CRC may be limited, emphasizing the potential for enhanced effectiveness through combination with other immune checkpoint inhibitors, such as anti-PD-L1.

#### 4.2.3 LAG-3 as an immunotherapeutic target: promising effect of favezelimab on CRC

Favezelimab is a humanized anti-LAG-3 IgG4 mAb that inhibits the binding between LAG-3 and its ligand, an MHC II molecule (215). It increases the production of cytokines (IFN-γ, IL-2, IL-8, and TNF-α) and chemokines (CCL4, CXCL10, and CCL22) in T cells. Similarly, it increases the expression of CD69, CD44, CD25, CXCL1, GZMB, and nuclear factor in activated T cells (216). Based on preliminary findings, favezelimab shows good safety and efficacy profiles as well as manageable tolerability when administered alone or in combination with other ICIs (217, 218). The safety and efficacy of this drug are being evaluated in a phase I/II first-in-human clinical trial in combination with pembrolizumab, an anti-PD-1 mAb (219). In an active phase III clinical trial (NCT05064059), the efficacy of anti-LAG-3 mAbs in CRC treatment is being assessed. Preliminary findings indicate that favezelimab alone or in combination with pembrolizumab has a manageable safety profile with no treatment-related deaths. Such promising data may open a window for new investigations into single or combined anti-LAG-3 treatments.

## 5 Conclusion

CRC treatment is on the verge of a transformative era owing to the development and application of ICIs such as PD-1/PD-L1 and CTLA-4 antibodies. These groundbreaking therapies offer hope for treating advanced stages of a disease that was once considered almost impossible to treat. A wealth of data from various clinical trials paints a complex yet promising landscape of treatment options, providing substantial hope for enhancing the prognosis of patients with CRC. CAR-T therapies, initially designed for blood cancers, show promise in treating CRC, but challenges such as the cytokine release syndrome and neurotoxicity highlight the need for ongoing innovation to improve their safety and effectiveness. Research into CAR-modified T cells indicates the potential of this modality in shrinking solid tumors but emphasizes the imperative for further advancements to amplify response rates and reduce risks. The inherent diversity within the context of CRC, as evidenced by varying responses to immunotherapies based on factors such as mismatch repair status or microsatellite instability, underscores the critical role of precision medicine. This calls for the development of individualized treatment strategies that are fine-tuned to the unique genetic and molecular characteristics of each patient’s tumor. Combination therapies that blend checkpoint inhibitors with vaccines, chemotherapies, or other immune-modifying agents could be more effective in stimulating an antitumor response than single-agent treatments. However, these complex approaches also introduce the challenge of identifying the most effective combinations, sequences, and dosages, which will be an important focus for future research endeavors. The future of CRC treatment lies in deepening the understanding of the TME and the dynamic relationship between cancer cells and the immune system. Identifying biomarkers that predict response and understanding the mechanisms behind resistance to treatment are crucial steps toward developing more effective therapeutic strategies. The next phase in this journey will involve leveraging the full capabilities of immunotherapies through innovative combinations and treatment planning while carefully managing potential side effects. In summary, while remarkable progress has been made in CRC treatment, there remains a pressing need for ongoing research and clinical development. Incorporating novel immunotherapeutic agents, refining existing treatments, and gaining a more nuanced understanding of CRC at a molecular level will contribute to more efficient, personalized treatment options. Persistently challenging existing paradigms and pushing the frontiers of scientific research hold the potential to transform CRC into a chronic condition that can be managed effectively and, perhaps eventually, lead to a cure for this challenging disease.

## Author contributions

SS: Writing – original draft, Writing – review & editing. NB: Writing – original draft. MD: Writing – original draft. HN: Writing – original draft. RA: Writing – original draft. JB: Writing – review & editing. AH: Conceptualization, Supervision, Writing – review & editing. AM: Conceptualization, Supervision, Writing – review & editing.
