# Supplementary material for: Redefining the battle against colorectal cancer: a comprehensive review of emerging immunotherapies and their clinical efficacy
Source: Front Immunol. 2024 Mar 12;15:1350208. doi: 10.3389/fimmu.2024.1350208 (PMC10963412; doi:10.3389/fimmu.2024.1350208)
Supplement: Supplementary file 1 [file Table_1.docx]

**Supplementary Table 1- Completed clinical trials using ICI in CRC.**

| **Intervention and combination treatment** | **Trial Title** | **Phase** | **Actual Enrollment (# of participants)** | **Main Findings** | **NCT number** | **Year/ Actual Study Completion Date** | **Ref.** |
| --- | --- | --- | --- | --- | --- | --- | --- |
| Pembrolizumab  mFOLFOX6 | Study of Pembrolizumab in Combination With Chemotherapy for Patients With Advanced Colorectal Cancer | II | 30 | T cells and myeloid cells did not influence the treatment outcomes, and this combination did not yield a superior anti-tumor effect compared to previously tested ones.  Further research utilizing a more effective combination could be achieved by focusing on biomarkers associated with positive treatment responses. | NCT02375672 | October 8, 2020 | (1) |
| Cyclophosphamide  GVAX  Pembrolizumab | Study of GVAX (With CY) and Pembrolizumab in MMR-p Advanced Colorectal Cancer | II | 17 | The combination fell short of the initial objectives in MMR-p CRC.  However, an improvement in the anti-tumor effect was observed compared to pembrolizumab treatment alone, possibly caused by GVAX. | NCT02981524 | March 20, 2018 | (2) |
| Pembrolizumab  Azacitidine | A Phase 2 Study of Pembrolizumab (MK-3475) in Combination With Azacitidine in Subjects With Chemo-refractory Metastatic Colorectal Cancer | II | 31 | This combination demonstrated reasonable disease control and a safe, tolerable effect in chemotherapy-refractory mCRC.  It is suggested that tumor immunomodulation is positively correlated with the use of the DNA demethylation agent azacitidine | NCT02260440 | September 2017 | (3) |
| Oral CC-486 (5-azacitidine)  Romidepsin  MK-3475 | A Study of Enhancing Response to MK-3475 in Advanced Colorectal Cancer | I | 27 | The combination of Pembrolizumab with 5-azacitidine and romidepsin was found to be well-tolerated in patients with MMR-proficient CRC.  Further research is required to determine the regimen's predictive responses both before and after treatment | NCT02512172 | November 20, 2021 | (4) |
| Cetuximab  Pembrolizumab | Cetuximab and Pembrolizumab in Treating Patients With Colorectal Cancer That is Metastatic or Cannot Be Removed by Surgery | I & II | 45 | This regimen modified the TME by recruiting CTL and expanding their population, as evidenced by the observed reduction in their numbers in PB. There was a noticeable decrease in PD1+ expressing cells within the TME, supported by an increase in CD4+CTLA4+ T-cells in peripheral blood. However, the results are not yet conclusive, and further research using clinical data is necessary to gain better insights | NCT02713373 | July 20, 2021 | (5) |
| Pembrolizumab | Study of Pembrolizumab (MK-3475) as Monotherapy in Participants With Previously-Treated Locally Advanced Unresectable or Metastatic Colorectal Cancer (MK-3475-164/KEYNOTE-164) | II | 124 | Pembrolizumab is effective and safe in treating MSI-CRC | NCT02460198 | February 19, 2021 | (6) |
| Pembrolizumab  Ibrutinib | Pembrolizumab in Combination With Ibrutinib for Advanced, Refractory Colorectal Cancers | I & II | 40 | This combination was found to be tolerated, but it exhibited low anti-tumor activity in mCRC | NCT03332498 | September 9, 2021 | (7) |
| Pembrolizumab  Maraviroc | Combined PD-1 and CCR5 Inhibition for the Treatment of Refractory Microsatellite Stable mCRC | I | 20 | -In this heavily pretreated cohort, the effectiveness of poststudy salvage treatment exceeded expectations.  -The combination of pembrolizumab and maraviroc proved to be safe, with a positive toxicological impact and stabilized MMR CRC diesase. does this paragraph mean the same as the above one | NCT03274804 | March 1, 2020 | (8) |
| Olaptesed (NOX-A12)  Pembrolizumab  Combination Therapy | Olaptesed (NOX-A12) Alone and in Combination With Pembrolizumab in Colorectal and Pancreatic Cancer | I & II | 20 | This combination has a safety record comparable to pembrolizumab in advanced cancer. The regimen induced an immune response in 25% of the patients and led to a prolonged treatment duration compared to prior treatments in 35% of the patients | NCT03168139 | March 25, 202 | (9) |
| AMG820 and pembrolizumab | Safety and Efficacy Study of AMG 820 and Pembrolizumab Combination in Select Advanced Solid Tumor Cancer | I & II | 117 | This regimen demonstrated a favorable safety profile at a dose of 1100 mg for AMG 820 and 200 mg for pembrolizumab. However, due to the low anti-tumor activity observed with this combination, further research does not appear promising | NCT02713529 | May 17, 2019 | (10) |
| MK-3475  (Pembrolizumab) | Study of MK-3475 in Patients With Microsatellite Unstable (MSI) Tumors (Cohorts A, B and C) | II | 113 | The treatment of mismatch instable tumors with immune checkpoint blockade, Pembrolizumab, is highly beneficial as it leads to an increase in progression-free survival.  Disease control rate (DCR) is 90% in dMMR.  At week 20, the progression-free survival (PFS) was 78% in dMMR, but overall survival (OS) had not been reached in this cohort | NCT01876511 | August 2019 | (11,12) |
| Epacadostat in combination with Immunotherapeutic drugs (including Pembrozulimab  ) and chemotherapies | A Study of Epacadostat in Combination With Pembrolizumab and Chemotherapy in Participants With Advanced or Metastatic Solid Tumors (ECHO-207/KEYNOTE-723) | I & II | 70 | The concurrent use of these drugs, pembrolizumab and chemotherapy, with Epacadostat administered at a dosage of 100 mg twice daily, showed a favorable safety profile.  Results also indicated that this treatment is effective against advanced and metastatic tumors. | NCT03085914 | July 13, 2020 | (13) |
| Avelumab | Avelumab and Cetuximab in Combination With FOLFOX in Patients With Previously Untreated Metastatic Colorectal Cancer - The Phase II AVETUX-CRC Trial. | II | 43 | A 79.5% objective response rate (ORR) to estimated time of surgery (ETS) ratio is promising for further investigations. The AVETUX regimen is feasible and produces a significant response, especially within the first 8 weeks, in MSS patients.  Through central radiological review, TIL clonality and diversity can be used as possible indicators of the response to the combination of chemotherapy and immunotherapy. | NCT03174405 | July 16, 2021 | (14,15) |
| eFT508  Avelumab | A Study to Evaluate eFT508 Alone and in Combination With Avelumab in Subjects With MSS Colorectal Cancer | II | 56 | This combination is safe, with a strong target interaction initially, and the results show some level of effectiveness. | NCT03258398 | May 13, 2019 | (16) |
| Avelumab  Cetuximab | Avelumab Plus Cetuximab in Pre-treated RAS Wild Type Metastatic Colorectal Cancer | II | 77 | In RAS wild-type mCRC patients, this combination is effective and well tolerated. Patients who can benefit from this treatment can be identified using plasma ctDNA analysis. | NCT04561336 | November 14, 2021 | (17) |
| Avelumab | Avelumab Plus Autologous Dendritic Cell Vaccine in Pre-treated Metastatic Colorectal Cancer Patients | I & II | 28 | This study represents a novel therapeutic approach that sensitizes tumors through therapeutic-induced metabolic reprogramming. The combination of Avelumab plus the ADC (Autologous dendritic cells) vaccine is safe, well tolerated, but shows limited clinical efficacy. | NCT03152565 | October 5, 2020 | (18) |
| Temozolomide  Nivolumab  Ipilimumab | Nivolumab Plus IPILIMUMAB and TEMOZOLOMIDE in Microsatellite Stable, MGMT Silenced Metastatic Colorectal Cancer (MAYA) | II | 135 | A significant clinical effect is to be seen upon priming with temozolomide followed by a combination of low-dose ipilimumab and nivolumab in this proof of concept study in MSS and MGMT-silenced mCRC. | NCT03832621 | September 30, 2021 | (19) |
| Nivolumab  Oxaliplatin  Leucovorin  Fluorouracil  Bevacizumab | An Investigational Immunotherapy Study of Nivolumab With Standard of Care Therapy vs Standard of Care Therapy for First-Line Treatment of Colorectal Cancer That Has Spread (CheckMate 9X8) | II & III | 195 | In mCRC, the combination of NIVO with SOC has been determined to be safe. There was an increase in PFS after 12 months and long-lasting disease control compared to SOC alone. | NCT03414983 | December 28, 2022 | (20) |
| Liver radiation therapy  Nivolumab Injection  Ipilimumab Injection [Yervoy]  CMP-001 | Combined Immunotherapy and Radiosurgery for Metastatic Colorectal Cancer | I | 19 | Intolerable liver toxicity is caused by this combination, intratumoral vidutolimod, radiosurgery, nivolumab, and ipilimumab,; hence, this combination is not effective. | NCT03507699 | May 25, 2022 | (21) |
| Biological: GRT-C901  Biological: GRT-R902  Biological: nivolumab  Biological: ipilimumab | A Study of a Personalized Neoantigen Cancer Vaccine | I & II | 214 | This study has two endpoints. The first endpoint demonstrated that the regimen is safe and tolerable, with no dose-dependent toxicity observed. Regarding the second endpoint, the vaccine elicited a CD8+ T cell response and improved overall survival (OS) in some patients with MSS-CRC. Consequently, these results warrant further research to explore this regimen | NCT03639714 | November 10,2022 | (22) |
| Drug: Based on sensitivity analysis | Predictive Value of Drug Sensitivity Testing Tumorspheres From Patients With Metastatic Colorectal Cancer | II | 90 | In mCRC patients, in vitro responsiveness and patient-specific organoids were feasible. No disease progression was observed in half of the patients for 2 months, meeting the first endpoint. This suggests the potential benefits of functional testing using tumor-derived organoids for cancer patient. | NCT03251612 | March 21,2022 | (23) |
| Drug: Part 1 TPST-1120  Drug: Part 2 TPST-1120 + nivolumab  Drug: Part 3 TPST-1120  Drug: Part 4 TPST-1120 + nivolumab | TPST-1120 as Monotherapy and in Combination With Nivolumab in Subjects With Advanced Cancers | I | 138 | The treatment alone or in combination has shown to be well tolerated and measurable improvement or reduction in the size or activity of tumors in the combination setting including responders in late-line RCC and heavily pretreated CCA, resistant to anti-PD-1 therapy. | NCT03829436 | September 7, 2022 | (24) |
| Regorafenib (Stivarga, BAY73-4506)  Nivolumab (Opdivo) | Study on the Effectiveness and Safety of the Combination of the Two Drugs Regorafenib and Nivolumab in Patients With Colorectal Cancer (Cancer of the Colon or Rectum Classified as Proficient Mismatch Repair and Microsatellite Stable) | II | 70 | For this combination to be more effective, further research is warranted to discover patients subgroups with biomarkers or clinical traits. | NCT04126733 | March 28, 2022 | (25) |
| TAS-102  nivolumab | A Study Evaluating TAS-102 Plus Nivolumab in Patients With MSS CRC | II | 18 | Although the combination showed to be feasible and tolerable, no therapeutic effect was seen in MSS-CRC. | NCT02860546 | September 7, 2017 | (26) |
| enadenotucirev  nivolumab | Phase I Study of Enadenotucirev and PD-1 Inhibitor in Subjects With Metastatic or Advanced Epithelial Tumors (SPICE) | I | 51 | This combinatorial regimen has been shown to have enduring tolerability, enhance immune cell tumor infiltration, and improve OS. | NCT02636036 | October 8, 2021 | (27) |
| Combination of varlilumab and nivolumab | A Dose Escalation and Cohort Expansion Study of Anti-CD27 (Varlilumab) and Anti-PD-1 (Nivolumab) in Advanced Refractory Solid Tumors | I & II | 175 | The combination is well tolerated with minimal side effects. While there was no significant response observed overall, there was a notable response in patients refractory to anti-PD-1 therapy. Treatment resulted in changes in the tumor microenvironment in ovarian cancer patient. | NCT02335918 | December 12, 2018 | (28) |
| Drug: Nivolumab (Phase 1)  Drug: Epacadostat (Phase 1)  Drug: Chemotherapy (Phase 1)  Drug: Nivolumab (Phase 2)  Drug: Epacadostat (Phase 2) | A Study of the Safety, Tolerability, and Efficacy of Epacadostat Administered in Combination With Nivolumab in Select Advanced Cancers (ECHO-204) | I & II | 307 | This combination is well tolerated and shows potential anti-cancer activity in melanoma patients. However, no final data have been provided for this study | NCT02327078 | July 10, 2020 | (29) |
| Combination of NKTR-214 + nivolumab | A Dose Escalation and Cohort Expansion Study of NKTR-214 in Combination With Nivolumab and Other Anti-Cancer Therapies in Patients With Select Advanced Solid Tumors (PIVOT-02) | I & II | 557 | BEMPEG plus nivolumab was well endured with antitumor activity as first-line treatment in patients with locally advanced/mUC. | NCT02983045 | April 28, 2022 | (30) |
| INCAGN01876  Nivolumab  Ipilimumab | Phase 1/2 Study Exploring the Safety, Tolerability, and Efficacy of INCAGN01876 Combined With Immune Therapies in Advanced or Metastatic Malignancies | I & II | 145 | INCAGN01876, in combination with NIVO and/or IPI treatments, was well tolerated with no major toxicity observed. The recommended phase 2 dose for INCAGN01876 is 300 mg administered every two weeks (Q2W) | NCT03126110 | November 9, 2021 | (31) |
| Talimogene Laherparepvec  Atezolizumab | Study of Talimogene Laherparepvec With Atezolizumab for Triple Negative Breast Cancer and Colorectal Cancer With Liver Metastases | I | 36 | No unprecedented safety issues were recorded after treatment with T-Vec or after the combination with atezolizumab. Low anti-tumor activitiy was observed. | NCT03256344 | December 3, 2021 | (32) |
| Atezolizumab (MPDL3280A), an Engineered Anti-PDL1 Antibody  Cobimetinib  Regorafenib | A Study to Investigate Efficacy and Safety of Cobimetinib Plus Atezolizumab and Atezolizumab Monotherapy Versus Regorafenib in Participants With Metastatic Colorectal Adenocarcinoma (COTEZO IMblaze370) | III | 363 | IMblaze370 did not meet its primary endpoint of improved overall survival with atezolizumab plus cobimetinib or atezolizumab versus regorafenib. The safety of atezolizumab plus cobimetinib was consistent with those of the individual drugs. These results underscore the challenge of expanding the benefit of immunotherapy to patients whose tumours have lower baseline levels of immune inflammation, such as those with microsatellite-stable metastatic colorectal cancer. | NCT02788279 | December 26, 2018 | (33) |
| Atezolizumab  Bevacizumab  Cobimetinib | Study of Cobimetinib in Combination With Atezolizumab and Bevacizumab in Participants With Gastrointestinal and Other Tumors | I | 51 | The combination is safe, and better outcomes were observed in patients with RAS mutations compared to those with wild-type genes | NCT02876224 | June 25, 2019 | (34) |
| Bevacizumab  Irinotecan  Oxaliplatin  L-Leucovorin  5-fluorouracil  Atezolizumab | FOLFOXIRI + Bev + Atezo vs FOLFOXIRI + Bev as First-line Treatment of Unresectable Metastatic Colorectal Cancer Patients | II | 201 | Combining atezolizumab to the initial regimen (FOLFOXIRI plus bevacizumab) is safe and escalated the PFS in previously untreated mCRC.  Survival improvement was observed in patients with high immune score (IS) pMMR mCRC using this combination as upfront treatment. The results need further investigation in a phase III study | NCT03721653 | August 31,2023 | (35,36) |
| Pexidartinib  Durvalumab | Evaluation of Safety and Activity of an Anti-PDL1 Antibody (DURVALUMAB) Combined With CSF-1R TKI (PEXIDARTINIB) in Patients With Metastatic/Advanced Pancreatic or Colorectal Cancers (MEDIPLEX) | I | 48 | Toxicity was consistent with the expected profiles of the individual drugs and no unexpected events were seen with the combination. Updated data will be presented at the meeting.  A restricted tumor response was observed, attributed to the use of the FLT3 inhibitor pexidartinib, which had a detrimental effect on dendritic cells (DCs). subsequently , FLT3 inhibition should be considered when combining tyrosine kinase inhibitors (TKIs) with anti-PD-L1 therapy | NCT02777710 | December 2019 | (37,38) |
| Cetuximab  FOLFOX induction regimen  Fluoropyrimidine (5-FU/LV or capecitabine) Atezolizumab  Vemurafenib  Bevacizumab  Trastuzumab  Pertuzumab  Cobimetinib  5-FU/LV | A Study of Biomarker-Driven Therapy in Metastatic Colorectal Cancer (mCRC) | II | 609 | Primary results suggested that in BRAF wild-type mCRC, PD-L1, CD8/GrB, and FoxP3 are not potential prognostic indicators. For this setting, further exploration is needed to determine the predictive indicators and factors.  In BRAFmut mCRC, Vemurafenib plus cetuximab plus 5-FU/LV merit further exploration and MAPK- pathway cutting edge genetic modification could offer a new therapeutic opportunity. Further combination should be tested in MSS mCRC as Cobimetinib plus atezolizumab showed undesirable results. | NCT02291289 | March 24,2021 | (39,40) |
| AZD4635  Durvalumab  Abiraterone Acetate  Enzalutamide  Oleclumab  Docetaxel | A Phase 1 Clinical Study of AZD4635 in Patients With Advanced Solid Malignancies | I | 313 | AZD4635 was well-tolerated both as a single agent and in combination therapy. In metastatic castration-resistant prostate cancer (mCRPC), further combinations warrant investigation in a phase II study. | NCT02740985 | March 31, 2023 | (41) |
| Durvalumab  Tremelimumab | Tremelimumab and Durvalumab in Treating Patients With Colorectal Cancer With Liver Metastases That Can Be Removed by Surgery | I | 22 | The activation of T and B cells was evident upon the use of this combination, and the safety level was apparent before liver resection in patients with pMMR mCRC | NCT02754856 | January 30,2023 | (42) |
| Standard of Care  Experimental  Chemotherapy and Bevacizumab | COLUMBIA-1: Novel Oncology Therapies in Combination With Chemotherapy and Bevacizumab as First- Line Therapy in MSS-CRC | I & II | 61 | This combination, consistent with the previous safety profile, has been deemed safe.  Following the addition of durvalumab and oleclumab to bevacizumab and FOLFOX (standard of care), a slight improvement was observed in comparison to the standard of care alone | NCT04068610 | October 10, 2022 | (43) |
| durvalumab  Tremelimumab | Study of Durvalumab and Tremelimumab After Radiation for Microsatellite Stable Metastatic Colorectal Cancer Progressing on Chemotherapy | II | 33 | The combination of two ICB with SBRT (palliative hypofractionated radiotherapy) was found to be safe and well-tolerated. In this regimen, two patients exhibited partial improvement, with response durations of 44 and 44+ weeks, in the context of treatment-resistant MSS CRC. | NCT03007407 | August 9, 2019 | (44) |
| durvalumab  tremelimumab  Radiation: Radiotherapy (RT)  Procedure: ablation | A Clinical Trial of Durvalumab and Tremelimumab, Administered with Radiation Therapy or Ablation in Patients with Colorectal Cancer | II | 25 | The study combination does not warrant further investigation after not meeting the predefined endpoints. However, the occurrence of an abscopal response in non-irradiated areas and a systemic escalation in the immune response is uncommon. In p-MSS CRC, combining durvalumab and tremelimumab is feasible with a tolerable safety profile. Future studies involving new combinations and predictive markers for abscopal responses are required. | NCT03122509 | April 28, 2021 | (45) |
| Durvalumab  (Anti-PD-L1)  Tremelimumab (Anti-CTLA-4) | A Pilot Feasibility Study of Yttrium-90 Liver Radioembolization Followed by Durvalumab and Tremelimumab in Patients with Microsatellite Stable Colorectal Cancer Liver Metastases | II | 9 | No tumor-guided immune response was noticed in MSS CRC that is metastasized to liver; however, Y90 radioembolization can be harmlessly combined with durvalumab and tremelimumab | NCT03005002 | November 26, 2019 | (46) |
| MEDI4736 | Evaluate the Efficacy of MEDI4736 in Immunological Subsets of Advanced Colorectal Cancer | II | 16 | In patient with MSI CRC, durvalumab was found to have a manageable safety profile, anti-tumor activity, and favorable overall survival. | NCT02227667 | June 29, 2020 | (47) |
| Tremelimumab  Durvalumab  Other: Best Supportive Care | Durvalumab and Tremelimumab and Best Supportive Care vs Best Supportive Care in Patients With Advanced Colorectal Cancer | II | 180 | In advanced unresponsive CRC, prolonged overall survival could be associated with the combination of durvalumab plus tremelimumab. Patients who can benefit from this regimen are to be identified by elevated plasma levels of tumor mutation burden (TMB).  Enhanced OS is more prominent in CMS2 than CMS4 when using this combination. The immune characteristics specific to each CMS type are crucial in determining the appropriate approach for targeting the desired immune regulation. | NCT02870920 | June 7, 2022 | (48,49) |
| Durvalumab  Trametinib | Study of Durvalumab (MEDI4736) (Anti-PD-L1) and Trametinib (MEKi) in MSS Metastatic Colon Cancer | II | 29 | In unresponsive mCRC, this combination demonstrated acceptable tolerability. However, the study will not progress to stage 2 due to the response rate not meeting the effectiveness benchmarks in stage 1. | NCT03428126 | May 5, 2022 | (50) |
| ONCOS-102  Durvalumab  Cyclophosphamide | A Study to Investigate ONCOS-102 in Combination With Durvalumab in Subjects With Advanced Peritoneal Malignancies | I & II | 67 | Combining durvalumab with IP ONCOS-102 was proven to be safe with no dose-limiting toxicity. Both biological and clinical activities were noticeable from preliminary evaluations.  The study did not meet its efficacy endpoint; however, the combination was well-tolerated. | NCT02963831 | June 25, 2022 | (51) |
| Azacitidine  Durvalumab | Study of Azacitidine and Durvalumab in Advanced Solid Tumors | II | 28 | In immunologically cold tumor, this combination did not show any pharmacological or clinical effect. This study supports further drug developments and studies to be done using these drugs. | NCT02811497 | August 4, 2020 | (52) |
| Durvalumab  Tremelimumab | A Phase 1 Study to Evaluate MEDI4736 in Combination With Tremelimumab | I | 104 | The combination regimen of Durva and Treme demonstrated a tolerable safety profile with indications of clinical effectiveness. These results warrant further investigation of this combination. | NCT01975831 | July 2, 2021 | (53) |
| Ticilimumab (CP-675,206)  Anti-CTLA-4 | Phase 2, Single Arm Study Of Ticilimumab In Patients With Refractory Metastatic Adenocarcinoma Of The Colon Or Rectum | Phase 2 | 47 | Despite the survival of 21 patients for more than six months and the interesting moderate response of one patient, Ticilimumab has shown no significant activity. In combination with other ICI, it could be promising. | NCT00313794 | June 2008 | (54) |
| XmAb20717 PD-1 x CTLA-4 Bispecific antibody | A Phase 1 Multiple Dose Study to Evaluate the Safety and Tolerability of XmAb®20717 in Subjects With Selected Advanced Solid Tumors | Phase 1 | 150 | This bispecific antibody is safe and showed pharmacodynamic activity in patients with advanced solid tumors who have undergone intensive pretreatment. | NCT03517488 | September 6, 2022 | (55) |

1. Herting CJ, Farren MR, Tong Y, Liu Z, O'Neil B, Bekaii-Saab T, et al. A multi-center, single-arm, phase Ib study of pembrolizumab (MK-3475) in combination with chemotherapy for patients with advanced colorectal cancer: HCRN GI14-186. Cancer Immunol Immunother. 2021;70(11):3337-48.

2. Yarchoan M, Huang CY, Zhu Q, Ferguson AK, Durham JN, Anders RA, et al. A phase 2 study of GVAX colon vaccine with cyclophosphamide and pembrolizumab in patients with mismatch repair proficient advanced colorectal cancer. Cancer Med. 2020;9(4):1485-94.

3. Kuang C, Park Y, Augustin RC, Lin Y, Hartman DJ, Seigh L, et al. Pembrolizumab plus azacitidine in patients with chemotherapy refractory metastatic colorectal cancer: a single-arm phase 2 trial and correlative biomarker analysis. Clin Epigenetics. 2022;14(1):3.

4. Murphy AG, Walker R, Lutz ER, Parkinson R, Ahuja N, Zheng L, et al. Epigenetic priming prior to pembrolizumab in mismatch repair-proficient advanced colorectal cancer. Journal of Clinical Oncology. 2019;37(4_suppl):591-.

5. Boland PM, Muhitch J, Abrams SI, Maguire O, Minderman H, Bajor DL, et al. Initial correlative studies from a trial of cetuximab and pembrolizumab in metastatic colorectal cancer (mCRC). Journal of Clinical Oncology. 2020;38(15_suppl):4062-.

6. Le DT, Kim TW, Van Cutsem E, Geva R, Jager D, Hara H, et al. Phase II Open-Label Study of Pembrolizumab in Treatment-Refractory, Microsatellite Instability-High/Mismatch Repair-Deficient Metastatic Colorectal Cancer: KEYNOTE-164. J Clin Oncol. 2020;38(1):11-9.

7. Kim DW, Tan E, Zhou JM, Schell MJ, Martinez M, Yu J, et al. A phase 1/2 trial of ibrutinib in combination with pembrolizumab in patients with mismatch repair proficient metastatic colorectal cancer. Br J Cancer. 2021;124(11):1803-8.

8. Haag GM, Springfeld C, Grun B, Apostolidis L, Zschabitz S, Dietrich M, et al. Pembrolizumab and maraviroc in refractory mismatch repair proficient/microsatellite-stable metastatic colorectal cancer - The PICCASSO phase I trial. Eur J Cancer. 2022;167:112-22.

9. Halama N, Prüfer U, Froemming A, Beyer D, Eulberg D, Jungnelius JU, et al. 613P - Phase I/II study with CXCL12 inhibitor NOX-A12 and pembrolizumab in patients with microsatellite-stable, metastatic colorectal or pancreatic cancer. Annals of Oncology. 2019;30:v231.

10. Razak AR, Cleary JM, Moreno V, Boyer M, Calvo Aller E, Edenfield W, et al. Safety and efficacy of AMG 820, an anti-colony-stimulating factor 1 receptor antibody, in combination with pembrolizumab in adults with advanced solid tumors. J Immunother Cancer. 2020;8(2).

11. Le DT, Uram JN, Wang H, Bartlett BR, Kemberling H, Eyring AD, et al. PD-1 Blockade in Tumors with Mismatch-Repair Deficiency. N Engl J Med. 2015;372(26):2509-20.

12. Le DT, Durham JN, Smith KN, Wang H, Bartlett BR, Aulakh LK, et al. Mismatch repair deficiency predicts response of solid tumors to PD-1 blockade. Science. 2017;357(6349):409-13.

13. Powderly JD, Klempner SJ, Naing A, Bendell J, Garrido-Laguna I, Catenacci DVT, et al. Epacadostat Plus Pembrolizumab and Chemotherapy for Advanced Solid Tumors: Results from the Phase I/II ECHO-207/KEYNOTE-723 Study. Oncologist. 2022;27(11):905-e848.

14. Stein A, Binder M, Goekkurt E, Lorenzen S, Riera-Knorrenschild J, Depenbusch R, et al. Avelumab and cetuximab in combination with FOLFOX in patients with previously untreated metastatic colorectal cancer (MCRC): Final results of the phase II AVETUX trial (AIO-KRK-0216). Journal of Clinical Oncology. 2020;38(4_suppl):96-.

15. Tintelnot J, Ristow I, Sauer M, Simnica D, Schultheiss C, Scholz R, et al. Translational analysis and final efficacy of the AVETUX trial - Avelumab, cetuximab and FOLFOX in metastatic colorectal cancer. Front Oncol. 2022;12:993611.

16. Hubbard JM, Patel MR, Bekaii-Saab TS, Falchook GS, Freilich BL, Dasari A, et al. A phase II, open label, randomized, noncomparative study of eFT508 (tomivosertib) alone or in combination with avelumab in subjects with relapsed/refractory microsatellite stable colorectal cancer (MSS CRC). Journal of Clinical Oncology. 2019;37(15_suppl):e14145-e.

17. Martinelli E, Martini G, Famiglietti V, Troiani T, Napolitano S, Pietrantonio F, et al. Cetuximab Rechallenge Plus Avelumab in Pretreated Patients With RAS Wild-type Metastatic Colorectal Cancer: The Phase 2 Single-Arm Clinical CAVE Trial. JAMA Oncol. 2021;7(10):1529-35.

18. Espanol-Rego M, Fernandez-Martos C, Elez E, Foguet C, Pedrosa L, Rodriguez N, et al. A Phase I-II multicenter trial with Avelumab plus autologous dendritic cell vaccine in pre-treated mismatch repair-proficient (MSS) metastatic colorectal cancer patients; GEMCAD 1602 study. Cancer Immunol Immunother. 2023;72(4):827-40.

19. Morano F, Raimondi A, Pagani F, Lonardi S, Salvatore L, Cremolini C, et al. Temozolomide Followed by Combination With Low-Dose Ipilimumab and Nivolumab in Patients With Microsatellite-Stable, O(6)-Methylguanine-DNA Methyltransferase-Silenced Metastatic Colorectal Cancer: The MAYA Trial. J Clin Oncol. 2022;40(14):1562-73.

20. Lenz H-J, Parikh AR, Spigel DR, Cohn AL, Yoshino T, Kochenderfer MD, et al. Nivolumab (NIVO) + 5-fluorouracil/leucovorin/oxaliplatin (mFOLFOX6)/bevacizumab (BEV) versus mFOLFOX6/BEV for first-line (1L) treatment of metastatic colorectal cancer (mCRC): Phase 2 results from CheckMate 9X8. Journal of Clinical Oncology. 2022;40(4_suppl):8-.

21. Lawrence YR, Lieberman S, Redinsky I, Honig N, Shacham-Shmueli E, Halpern N, et al. Combination treatment of intratumoral vidutolimod (CMP-001), radiosurgery, nivolumab, and ipilimumab for metastatic colorectal carcinoma. Journal of Clinical Oncology. 2023;41(4_suppl):123-.

22. Palmer CD, Rappaport AR, Davis MJ, Hart MG, Scallan CD, Hong SJ, et al. Individualized, heterologous chimpanzee adenovirus and self-amplifying mRNA neoantigen vaccine for advanced metastatic solid tumors: phase 1 trial interim results. Nat Med. 2022;28(8):1619-29.

23. Jensen LH, Rogatto SR, Lindebjerg J, Havelund B, Abildgaard C, do Canto LM, et al. Precision medicine applied to metastatic colorectal cancer using tumor-derived organoids and in-vitro sensitivity testing: a phase 2, single-center, open-label, and non-comparative study. J Exp Clin Cancer Res. 2023;42(1):115.

24. Yarchoan M, Powderly JD, Bastos BR, Karasic TB, Crysler OV, Munster PN, et al. A phase 1 study of TPST-1120 as a single agent and in combination with nivolumab in subjects with advanced solid tumors. Journal of Clinical Oncology. 2022;40(16_suppl):3005-.

25. Fakih M, Raghav KPS, Chang DZ, Larson T, Cohn AL, Huyck TK, et al. Regorafenib plus nivolumab in patients with mismatch repair-proficient/microsatellite stable metastatic colorectal cancer: a single-arm, open-label, multicentre phase 2 study. EClinicalMedicine. 2023;58:101917.

26. Patel MR, Falchook GS, Hamada K, Makris L, Bendell JC. A phase 2 trial of trifluridine/tipiracil plus nivolumab in patients with heavily pretreated microsatellite-stable metastatic colorectal cancer. Cancer Med. 2021;10(4):1183-90.

27. Fakih M, Harb W, Mahadevan D, Babiker H, Berlin J, Lillie T, et al. Safety and efficacy of the tumor-selective adenovirus enadenotucirev, in combination with nivolumab, in patients with advanced/metastatic epithelial cancer: a phase I clinical trial (SPICE). J Immunother Cancer. 2023;11(4).

28. Sanborn RE, Pishvaian MJ, Callahan MK, Weise A, Sikic BI, Rahma O, et al. Safety, tolerability and efficacy of agonist anti-CD27 antibody (varlilumab) administered in combination with anti-PD-1 (nivolumab) in advanced solid tumors. J Immunother Cancer. 2022;10(8).

29. Daud A, Saleh MN, Hu J, Bleeker JS, Riese MJ, Meier R, et al. Epacadostat plus nivolumab for advanced melanoma: Updated phase 2 results of the ECHO-204 study. Journal of Clinical Oncology. 2018;36(15_suppl):9511-.

30. Siefker-Radtke AO, Cho DC, Diab A, Sznol M, Bilen MA, Balar AV, et al. Bempegaldesleukin plus Nivolumab in First-line Metastatic Urothelial Carcinoma: Results from PIVOT-02. Eur Urol. 2022;82(4):365-73.

31. Gutierrez M, Balmanoukian AS, Shields AF, Johnson ML, Ulahannan SV, Barve MA, et al. A phase 1/2 study of the safety, tolerability, and preliminary efficacy of the anti-GITR monoclonal antibody, INCAGN01876, combined with immunotherapies (IO) in patients (Pts) with advanced cancers. Journal of Clinical Oncology. 2023;41(16_suppl):2541-.

32. Hecht JR, Raman SS, Chan A, Kalinsky K, Baurain JF, Jimenez MM, et al. Phase Ib study of talimogene laherparepvec in combination with atezolizumab in patients with triple negative breast cancer and colorectal cancer with liver metastases. ESMO Open. 2023;8(2):100884.

33. Eng C, Kim TW, Bendell J, Argilés G, Tebbutt NC, Di Bartolomeo M, et al. Atezolizumab with or without cobimetinib versus regorafenib in previously treated metastatic colorectal cancer (IMblaze370): a multicentre, open-label, phase 3, randomised, controlled trial. Lancet Oncol. 2019;20(6):849-61.

34. Bendell J, Lieu C, Raghav KPS, Argilés G, Cubillo A, Qu X, et al. A phase Ib study of the safety and efficacy of atezolizumab (atezo) + bevacizumab (bev) + cobimetinib (cobi) in patients (pts) with metastatic colorectal cancer (mCRC). Annals of Oncology. 2019;30:v227-v8.

35. Antoniotti C, Rossini D, Pietrantonio F, Catteau A, Salvatore L, Lonardi S, et al. Upfront FOLFOXIRI plus bevacizumab with or without atezolizumab in the treatment of patients with metastatic colorectal cancer (AtezoTRIBE): a multicentre, open-label, randomised, controlled, phase 2 trial. The Lancet Oncology. 2022;23(7):876-87.

36. Antoniotti C, Rossini D, Pietrantonio F, Salvatore L, Marmorino F, Ambrosini M, et al. FOLFOXIRI plus bevacizumab and atezolizumab as upfront treatment of unresectable metastatic colorectal cancer (mCRC): Updated and overall survival results of the phase II randomized AtezoTRIBE study. Journal of Clinical Oncology. 2023;41(16_suppl):3500-.

37. Cassier PA, Garin G, Eberst L, Delord J-P, Chabaud S, Terret C, et al. MEDIPLEX: A phase 1 study of durvalumab (D) combined with pexidartinib (P) in patients (pts) with advanced pancreatic ductal adenocarcinoma (PDAC) and colorectal cancer (CRC). Journal of Clinical Oncology. 2019;37(15_suppl):2579-.

38. Aurélien V, Carlos G-R, Sylvie C, Céline R, Axelle N, Justine B, et al. The CSF-1R inhibitor Pexidartinib impacts dendritic cell differentiation through inhibition of FLT3 signaling and may antagonize the effect of durvalumab in patients with advanced cancer – results from a phase 1 study. medRxiv. 2023:2023.02.15.23285939.

39. Tabernero J, Grothey A, Arnold D, Ducreux M, O'Dwyer PJ, Perdicchio M, et al. Exploratory biomarker findings from cohort 2 of MODUL: An adaptable, phase 2, signal-seeking trial of fluoropyrimidine + bevacizumab ± atezolizumab maintenance therapy for BRAFwt metastatic colorectal cancer. Journal of Clinical Oncology. 2021;39(15_suppl):3570-.

40. Ducreux M, Tabernero J, Grothey A, Arnold D, O'Dwyer PJ, Gilberg F, et al. Clinical and exploratory biomarker findings from the MODUL trial (Cohorts 1, 3 and 4) of biomarker-driven maintenance therapy for metastatic colorectal cancer. Eur J Cancer. 2023;184:137-50.

41. Lim EA, Bendell JC, Falchook GS, Bauer TM, Drake CG, Choe JH, et al. Phase Ia/b, Open-Label, Multicenter Study of AZD4635 (an Adenosine A2A Receptor Antagonist) as Monotherapy or Combined with Durvalumab, in Patients with Solid Tumors. Clinical Cancer Research. 2022;28(22):4871-84.

42. Kanikarla Marie P, Haymaker C, Parra ER, Kim YU, Lazcano R, Gite S, et al. Pilot Clinical Trial of Perioperative Durvalumab and Tremelimumab in the Treatment of Resectable Colorectal Cancer Liver Metastases. Clin Cancer Res. 2021;27(11):3039-49.

43. Segal NH, Tie J, Kopetz S, Ducreux MP, Chen E, Dienstmann R, et al. 160P COLUMBIA-1: A phase Ib/II, open-label, randomized, multicenter study of durvalumab plus oleclumab in combination with chemotherapy and bevacizumab as first-line (1L) therapy in metastatic microsatellite-stable colorectal cancer (MSS-mCRC). Immuno-Oncology and Technology. 2022;16.

44. Lee JJ, Yothers G, George TJ, Fakih MG, Mallick AB, Mitchell EP, et al. Abstract 2257: Phase II study of dual immune checkpoint blockade (ICB) with durvalumab (Durva) plus tremelimumab (T) following palliative hypofractionated radiotherapy (SBRT) in patients (pts) with microsatellite-stable (MSS) metastatic colorectal cancer (mCRC) progressing on chemotherapy: NSABP FC-9. Cancer Research. 2019;79(13_Supplement):2257-.

45. Segal NH, Cercek A, Ku G, Wu AJ, Rimner A, Khalil DN, et al. Phase II Single-arm Study of Durvalumab and Tremelimumab with Concurrent Radiotherapy in Patients with Mismatch Repair-proficient Metastatic Colorectal Cancer. Clin Cancer Res. 2021;27(8):2200-8.

46. Wang C, Park J, Ouyang C, Longmate JA, Tajon M, Chao J, et al. A Pilot Feasibility Study of Yttrium-90 Liver Radioembolization Followed by Durvalumab and Tremelimumab in Patients with Microsatellite Stable Colorectal Cancer Liver Metastases. Oncologist. 2020;25(5):382-e776.

47. Segal NH, Wainberg ZA, Overman MJ, Ascierto PA, Arkenau H-T, Butler MO, et al. Safety and clinical activity of durvalumab monotherapy in patients with microsatellite instability–high (MSI-H) tumors. Journal of Clinical Oncology. 2019;37(4_suppl):670-.

48. Chen EX, Jonker DJ, Loree JM, Kennecke HF, Berry SR, Couture F, et al. Effect of Combined Immune Checkpoint Inhibition vs Best Supportive Care Alone in Patients With Advanced Colorectal Cancer: The Canadian Cancer Trials Group CO.26 Study. JAMA Oncol. 2020;6(6):831-8.

49. Loree JM, Topham JT, Kennecke HF, Feilotter H, Lee YS, Virk S, et al. Impact of consensus molecular subtyping (CMS) on survival in the CO.26 trial of durvalumab plus tremelimumab versus best supportive care (BSC) in metastatic colorectal cancer (mCRC). Journal of Clinical Oncology. 2022;40(16_suppl):3551-.

50. Johnson B, Haymaker CL, Parra ER, Soto LMS, Wang X, Thomas JV, et al. Phase II study of durvalumab (anti-PD-L1) and trametinib (MEKi) in microsatellite stable (MSS) metastatic colorectal cancer (mCRC). J Immunother Cancer. 2022;10(8).

51. Zamarin D, Odunsi K, Slomovitz B, Duska L, Nemunaitis J, Reilley M, et al. Phase I/II study to evaluate systemic durvalumab + intraperitoneal (IP) ONCOS-102 in patients with peritoneal disease who have epithelial ovarian (OC) or metastatic colorectal cancer (CRC): Interim phase I clinical and translational results. Journal of Clinical Oncology. 2020;38:3017-.

52. Taylor K, Loo Yau H, Chakravarthy A, Wang B, Shen SY, Ettayebi I, et al. An open-label, phase II multicohort study of an oral hypomethylating agent CC-486 and durvalumab in advanced solid tumors. J Immunother Cancer. 2020;8(2).

53. Callahan MK, Odunsi K, Sznol M, Nemunaitis J, Ott PA, Dillon P, et al. Abstract A006: Phase 1 study to evaluate the safety and tolerability of MEDI4736 (durvalumab, durva) + tremelimumab (treme) in patients with advanced solid tumors. Cancer Immunology Research. 2019;7(2_Supplement):A006-A.

54. Chung KY, Gore I, Fong L, Venook A, Beck SB, Dorazio P, et al. Phase II study of the anti-cytotoxic T-lymphocyte-associated antigen 4 monoclonal antibody, tremelimumab, in patients with refractory metastatic colorectal cancer. J Clin Oncol. 2010;28(21):3485-90.

55. Hickingbottom B, Clynes R, Desjarlais J, Li C, Ding Y. Preliminary safety and pharmacodynamic (PD) activity of XmAb20717, a PD-1 x CTLA-4 bispecific antibody, in a phase I dose escalation study of patients with selected advanced solid tumors. Journal of Clinical Oncology. 2020;38(15_suppl):e15001-e.
